# Early aortic valve intervention versus clinical surveillance in patients with asymptomatic severe aortic stenosis: a systematic review and meta-analysis

**DOI:** 10.3389/fcvm.2026.1830856

**Published:** 2026-06-23

**Authors:** Umar G. Adamu, Confidence Makgoro, El-ameen U. Adamu, David Mashilo, Anupa Patel, Nqoba Tsabedze

**Affiliations:** 1 Division of Cardiology, Department of Internal Medicine, School of Clinical Medicine, Faculty of Health Sciences, University of the Witwatersrand, Johannesburg, South Africa; 2 Department of Internal Medicine, Federal Teaching Hospital, Birnin Kebbi, Nigeria

**Keywords:** aortic valve replacement, asymptomatic, clinical surveillance, hospitalization for heart failure, major adverse cardiovascular events, severe aortic stenosis

## Abstract

**Background:**

The optimal timing of aortic valve replacement (AVR) in patients with asymptomatic severe aortic stenosis (AS) remains uncertain. The aim of this study was to evaluate the efficacy and safety of early AVR compared to clinical surveillance (CSV) in patients with asymptomatic severe AS.

**Methods:**

We systematically searched PubMed, Embase, Cochrane Library, Web of Science, and Scopus through August 2025 for studies that compared early AVR with conservative management in asymptomatic patients with severe AS. Odds ratios (ORs) with 95% confidence intervals (CIs) were calculated using random-effects models.

**Results:**

Eight studies including four randomized controlled trials and four propensity-matched observational studies, enrolling 3,086 patients, of whom 1,539 (49.9%) underwent early AVR were included. Compared with CSV, AVR was associated with significant reduction in MACE (OR: 0.41, 95% CI: 0.29–0.59; *P* < 0.001), all-cause mortality (OR: 0.59, 95% CI: 0.39–0.88; *P* = 0.011), cardiovascular mortality (OR: 0.49, 95% CI: 0.35–0.69; *P* < 0.001), hospitalization for heart failure (OR 0.36, 95% CI 0.20–0.65; *P* < 0.001), and sudden death (OR: 0.51, 95% CI: 0.29–0.88; *P* = 0.015). Major vascular bleeding was more frequent in the early AVR group than in the conservative surveillance group (OR: 1.75, 95% CI: 1.05–2.92; *P* = 0.032). The risk of myocardial infarction, stroke, pacemaker implantation, or infective endocarditis were similar between groups.

**Conclusion:**

In patients with asymptomatic severe aortic stenosis, an early AVR strategy was associated with reduced heart failure hospitalization, favorable composite and procedural safety outcomes compared with CSV. Although a survival benefit was observed in pooled analyses, this was not consistently demonstrated in randomized trials. These findings support a more individualized approach to the timing of intervention.

**Systematic Review Registration:**

https://www.crd.york.ac.uk/PROSPERO/view/ CRD420251135500.

## Introduction

Severe aortic stenosis (AS) is a progressive and life-threatening condition affecting approximately 5% of individuals over 65 years of age, with markedly poor outcomes once symptoms develop (1). Nonetheless, up to half of patients with severe AS are asymptomatic at the time of diagnosis (2, 3). The enhanced periprocedural safety and proven long-term efficacy of both surgical and transcatheter interventions underscore the importance of timely decision-making in identifying patients with asymptomatic severe AS who may benefit from earlier AVR (4, 5).

The management of these patients has traditionally relied on vigilant clinical and echocardiographic surveillance, with AVR deferred until the development of symptoms or left ventricular dysfunction, an approach accorded a Class IIa recommendation in both the European Society of Cardiology (ESC) and American College of Cardiology/American Heart Association (ACC/AHA) guidelines (6, 7). Evidence from randomized controlled trials (RCTs), observational studies, and meta-analyses indicates that early AVR provides substantial survival and hospitalization for heart failure benefits (8–14). However, recent randomized trials have yielded heterogeneous results. While the EVOLVED trial did not demonstrate a significant difference in its primary endpoint, interpretation of these findings requires caution given important methodological considerations, including delays in the intervention arm that may have attenuated the intended pre-emptive treatment strategy (15). In contrast, trials such as RECOVERY, AVATAR, and EARLY TAVR have demonstrated favorable outcomes with early intervention (8, 9, 16).

Several recent meta-analyses have evaluated early aortic valve intervention vs. CSV in asymptomatic severe AS, particularly following the publication of contemporary randomized trials (15, 16). However, important gaps remain. Prior syntheses have variably included heterogeneous observational data without consistent adjustment for baseline confounding and have provided limited comparative interpretation between randomized and real-world evidence. In addition, the increasing adoption of TAVR has introduced further complexity in interpreting outcomes across procedural eras. Few analyses have systematically evaluated patient classification, specifically whether individuals are truly asymptomatic and truly have severe disease or comprehensively synthesized procedural safety outcomes such as pacemaker implantation, infective endocarditis, and major vascular complications. Recently, additional propensity score–matched observational studies have been published, providing higher-quality real-world data that may complement randomized evidence (17). Accordingly, we performed an updated systematic review and meta-analysis integrating contemporary randomized and propensity-matched observational studies to better define the benefits, risks, and optimal timing of early intervention in patients with asymptomatic severe AS.

## Material and methods

The meta-analysis was prospectively registered with the International Prospective Register of Systematic Reviews (PROSPERO) (Registration number: CRD420251135500). The study was conducted in accordance with the methodological recommendations of the Cochrane Collaboration and reported following the Preferred Reporting Items for Systematic Reviews and Meta-Analyses (PRISMA) guideline (18).

### Search strategy

We systematically searched PubMed, Embase, Cochrane Central Register of Controlled Trials (CENTRAL), Web of Science, and Scopus from inception to August 2025 for studies published in English. The PubMed search strategy was as follows: [“Aortic stenosis"[Mesh] OR “aortic valve stenosis” OR “aortic valve stenosis"[Mesh] OR “aortic valve disease” OR “severe aortic stenosis”] AND [Asymptomatic OR “asymptomatic diseases"(Mesh) OR “no symptoms” OR “minimally symptomatic”] AND [“Early intervention” OR “early surgery” OR “early aortic valve replacement” OR “early AVR” OR “preemptive surgery” OR “timely intervention” OR “aortic valve replacement"[Mesh] OR TAVR OR TAVI OR “transcatheter aortic valve replacement” OR “Transcatheter Aortic Valve Replacement"[Mesh] OR “surgical aortic valve replacement”). U.G.A and E.U.A manually searched the references of included studies and previous reviews. The search strategy of all the electronic databases is provided in Supplementary Table S1.

### Eligibility criteria

We included studies that met the following eligibility criteria: (1) peer reviewed RCTs and non-randomized studies; (2) comparing early valve intervention with clinical surveillance; (3) in patients with asymptomatic severe AS; and (4) reporting at least one of the clinical outcomes of interest. We excluded studies with (1) no control group; (2) enrolled patients with indication for aortic valve intervention; (3) overlapping studies, letters, abstracts, or editorials. In case of overlapping population, we only included the study with the highest number of patients. The intervention group was defined as an early (pre-emptive) AVR strategy, whereas the comparator group consisted of an initial clinical surveillance strategy with valve intervention performed when clinically indicated.

### Data extraction and endpoints

Article screening and data extraction were undertaken independently by three reviewers (U.G.A., C.M, and E.U.A) using a standardized form. We extracted the following study characteristics: (1) author; (2) journal name; (3) year of publication; (4) sample size; (5) patient baseline characteristics; and (6) follow-up period. The endpoints of interest were: (1) major adverse cardiovascular events (MACE); (2) all-cause mortality; (3) cardiovascular mortality; (4) stroke; (5) hospitalization for heart failure; and (6) myocardial infarction. Additionally, the incidences of sudden death, pacemaker implantation, endocarditis, and major vascular bleeding were also assessed. Furthermore, we extracted study-level information regarding the assessment of asymptomatic status and the criteria used to define severe AS to explore potential sources of heterogeneity. The definition and diagnostic criteria for asymptomatic severe aortic stenosis, as well as the specific MACE components evaluated in each study, including a structured assessment of whether patients were “truly asymptomatic” and “truly severe” are summarized in Supplementary Table S2. The definition of MACE varied across studies, comprising combinations of all-cause mortality, myocardial infarction, stroke, and heart failure hospitalization, and in some cases including aortic valve replacement or cardiovascular hospitalization. Disagreements between authors were resolved by consensus and the senior author (D.M, A.P, and N.T) after checking the for any discrepancies. Data from the longest follow-up time available were extracted for analysis.

### Subgroup analyses

We performed prespecified subgroup analyses based on study design and procedural modality (SAVR-only vs. TAVR-including studies). Additional subgroup analyses based on MACE definitions, asymptomatic status, and disease severity were considered; however, these were not performed quantitatively due to heterogeneity in reporting and the limited number of studies. Instead, these variables were systematically evaluated qualitatively and summarized in Supplementary Table S2. Interaction between subgroups was formally assessed using a *χ*² test for subgroup differences, with a *P*-value <0.10 considered suggestive of potential effect modification, in accordance with the Cochrane Handbook for Systematic Reviews of Interventions (19).

### Quality assessment

We evaluated the risk of bias in cohort studies using the Risk of Bias in Non-randomized Studies-of Intervention (ROBINS-1) and with the Cochrane Collaboration's tools for assessing risk of bias in randomized trials (RoB2) (20, 21). Two independent authors (U.G.A. and C.M) performed the risk of bias assessment, with disagreements resolved by consensus or by discussion with other authors. The layout was produced by Robvis tool (22). Sensitivity analyses were conducted using a leave-one-out approach for all outcomes, sequentially excluding each study to evaluate the robustness of the pooled estimates and the influence of individual studies on heterogeneity. Publication bias was assessed visually using funnel plots and statistically using Egger's regression test and Begg's rank correlation test, where applicable.

### Statistical analysis

The DerSimonian and Laird random-effects model was used for all pooled analyses. For binary outcomes, odds ratios (ORs) with 95% confidence intervals (CIs) were calculated using the Mantel-Haenszel method as the primary effect measure. In addition, risk ratios (RRs) were performed as a *post hoc* sensitivity analysis to assess the robustness and interpretability of the findings. As a further *post hoc* sensitivity analysis, hazard ratios (HRs) with corresponding 95% CIs were synthesized for outcomes where published time-to-event estimates were available. Reported HRs were extracted directly from the original studies and pooled using the inverse-variance random-effects model, with logarithmic transformation of HRs and calculation of standard errors from the reported CIs. Heterogeneity was evaluated with Cochran's *Q* test, Higgins and Thompsons's *I*^2^ statistics, and Tau-square using the restricted maximum-likelihood estimator and reported as low (*I*^2^ = 0%–25%), moderate (*I*^2^ = 26%–50%), or significant (*I*^2^ > 50%). Absolute risk differences (ARD) and corresponding numbers needed to treat or harm (NNT/NNH) were calculated using pooled event rates to enhance clinical interpretability. These estimates were derived from aggregated study-level data and should therefore be considered approximate. To explore sources of significant heterogeneity, we performed exploratory meta-regression analyses on MACE, all-cause mortality, and heart failure hospitalization using key study-level variables, including study design (randomized vs. non-randomized), procedural modality (TAVR-including vs. SAVR-only), and follow-up duration. Given the limited number of included studies, only univariable meta-regression analyses were undertaken, with each covariate evaluated separately (e.g., study design, procedural modality, and follow-up duration) to minimize overfitting and in accordance with methodological recommendations. Also, *I*^2^ values, including *I*^2^ = 0% to 49% were interpreted cautiously, as statistical tests for heterogeneity may be underpowered and unable to detect true between-study variability. The certainty of evidence for each outcome was evaluated using the Grading of Recommendations Assessment, Development and Evaluation (GRADE) approach. RCTs were initially rated as high-certainty evidence and propensity score-matched observational studies as low-certainty evidence, with subsequent downgrading or upgrading based on predefined domains, including risk of bias, inconsistency, indirectness, imprecision, and publication bias. In analyses incorporating both randomized and observational studies, overall certainty ratings were determined by considering the relative contribution and consistency of evidence across study designs. GRADE assessments were performed at the outcome level. The guidelines of the Cochrane Handbook for Systematic Reviews of Interventions were used for data handling (19). Data conversions were performed with the Luo and Wan methods (23, 24). *P*-values of <0.05 were considered statistically significant. All statistical analyses were performed using R statistical software, version 4.5.2 (R Foundation for Statistical Computing) and the RevMan 5.4.1 for statistical analysis (Nordic Cochrane Centre, The Cochrane Collaboration, Copenhagen, Denmark).

## Results

### Study selection and baseline characteristics

**Figure 1:**
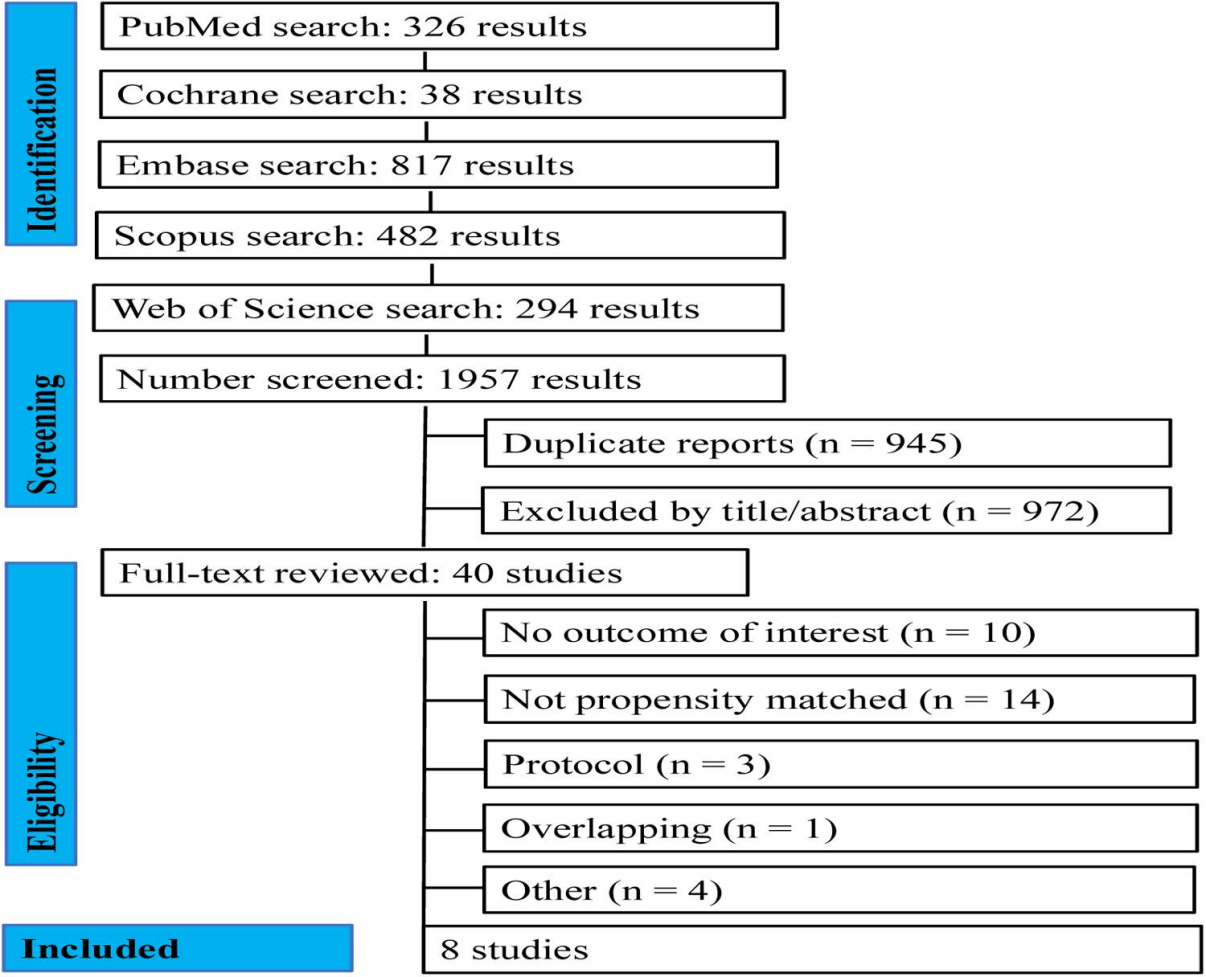
PRISMA Flow Diagram of Study Selection. Literature search and screening process for inclusion in the systematic review and meta-analysis. A total of 1,181 records were identified through PubMed (*n* = 326), Embase (*n* = 817), and Cochrane Library (*n* = 38). After removal of duplicates (*n* = 393), 788 records underwent title and abstract screening, of which 747 were excluded. Forty-one full-text articles were reviewed for eligibility, with exclusions for no outcome of interest (*n* = 10), lack of propensity matching (*n* = 14), protocols (*n* = 3), overlap (*n* = 1), and other reasons (*n* = 4). Finally, 8 studies met inclusion criteria and were included in the meta-analysis.

Our systematic search of electronic databases identified 1,957 records (Figure 1). After removal of duplicates and title/abstract screening, 40 studies were retrieved for full-text review. Of these, four RCTs and four observational studies fulfilled the inclusion criteria and were incorporated into both qualitative and quantitative analyses [8–11, 14–17]. In total, 3,086 patients were included, of whom 1,539 (49.9%) underwent early AVR. The mean age across studies ranged from 63 to 80 years, and 54.5% of patients were male. The follow-up period ranged from index hospitalization to 6 years. The Society of Thoracic Surgeons Predicted Risk of Mortality (STS-PROM) score ranged from 1.7% to 3.3%, while the mean aortic valve area at baseline ranged from 0.6 to 0.9 cm². Notably, Loganath et al. was the only study that used mid-wall late gadolinium enhancement (LGE) on cardiac magnetic resonance as an inclusion criterion [15]. Surgical AVR was exclusively performed in half of the included studies [8–10,14], TAVR in the EARLY TAVR trial [16], and a combination of SAVR and TAVR in four studies [11, 15, 17]. The baseline characteristics of included studies are summarized in Table 1. The variability in the assessment of asymptomatic status and definition of severe AS across studies is summarized in Supplementary Table S2, including a structured evaluation of whether patients were “truly asymptomatic” and “truly severe.”

**Table 1:** Baseline characteristics of included studies.

	AVATAR 2024 SAVR		EARLY TAVR 2024 TAVR		EVOLVED 2024 TAVR & SAVR		Kang 2010 SAVR		Kim 2019 TAVR & SAVR		RECOVERY 2020 SAVR		Takeji 2025 TAVR & SAVR		Taniguchi 2015 TAVR & SAVR	
Study design	RCT		RCT		RCT		Observational		Observational		RCT		Observational		Observational	
Country	Europe		US & Canada		UK & Australia		Korea		Korea		Korea		Japan		Japan	
Group	AVR	CS	AVR	CS	AVR	CS	AVR	CS	AVR	CS	AVR	CS	AVR	CS	AVR	CS
Sample size (*n*)	*n* = 78	*n* = 79	*n* = 455	*n* = 446	*n* = 113	*n* = 111	*n* = 102	*n* = 95	*n* = 221	*n* = 247	*n* = 73	*n* = 72	*n* = 206	*n* = 206	*n* = 291	*n* = 291
Age, years^‡^	63	69.4	76	75.6	73.9	74.6	63	63	61	67	65	63.3	79	80	71.6	73.1
Male, *n*	46	44	324	299	82	79	55	44	110	126	37	34	78	75	126	124
BMI, Kg/m^2^^‡^	NA	NA	28.4	28.6	27.6	27.9	23.9	24.1	24.6	23.6	24.7	24	22.8	23	22.1	22.9
Hypertension, *n*	69	70	369	365	76	70	37	39	92	122	40	39	168	177	188	187
Diabetes mellitus, *n*	14	23	119	114	15	26	10	10	42	77	13	7	NA	NA	59	66
PAD, *n*	0	1	33	21	4	9	NA	NA	2	4	1	2	19	9	28	31
Smoking, *n*	NA	NA	NA	NA	51	55	26	23	NA	NA	19	21	47	64	22	25
Previous PCI, *n*	1	2	NA	NA	7	7	NA	NA	7	13	3	1	1	1	21	31
Previous CABG, *n*	NA	NA	NA	NA	3	3	NA	NA	NA	NA	NA	NA	3	1	7	7
STS-PROM score, (%)^‡^	1.7	1.9	1.8	1.7	NA	NA	NA	NA	NA	NA	NA	NA	3.3	3.3	2.2	2.7
LVEF, (%)*^‡^*	NA	NA	67.4	67.4	68	68	62	63	63.7	63.1	64.8	64.8	65	66	66.8	68.2
AV peak velocity, m/sec^‡^	4.5	4.4	4.3	4.4	4.3	4.4	5.1	4.9	4.7	4.5	5.1	5.0	4.6	4.4	4.8	4.4
Mean AV PG, mmHg^‡^	51	50.7	46.5	47.3	45.2	45	65	59	55	64.3	62.7	48.6	48.1	45.3	54	45
AV area, cm^2^^‡^	0.71	0.74	0.9	0.8	0.8	0.8	0.6	0.62	0.74	0.64	0.64	0.8	0.7	0.8	0.67	0.75
Bicuspid AV, n^†^	NA	NA	37	39	36	28	57	39	127	49	39	63	24	16	53^†^	33^†^
Follow-up	63*		3.8^#^		42*		1,265^!^ (AVR) 1,769^!^ (CSV)		60.9*		6.2^#^ (AVR) 6.1^#^ (CSV)		3^#^		1,361^!^	

AV, aortic valve; AVR, aortic valve replacement; BMI, body-mass index; CABG, coronary artery bypass graft; CS, clinical surveillance; LVEF, left ventricular ejection fraction; NA, not available; PAD, peripheral arterial disease; PG, pressure gradient; RCT, randomized controlled trials; TAVR, transcatheter aortic valve replacement; SAVR, surgical aortic valve replacement; STS-PROM score, the society of thoracic surgeons predicted risk of mortality score; UK, United Kingdom; US, United states.

Summary of randomized controlled trials (RCTs) and propensity-matched observational studies evaluating early aortic valve replacement (AVR) versus conservative surveillance (CSV) in patients with asymptomatic severe aortic stenosis. The table includes study design, country, sample size, type of intervention (surgical AVR (SAVR) or transcatheter AVR (TAVR), baseline demographics (age, sex, BMI), cardiovascular risk factors [hypertension, diabetes mellitus, peripheral arterial disease (PAD), smoking], prior coronary interventions [percutaneous coronary intervention (PCI), coronary artery bypass grafting (CABG)], surgical risk profile [Society of Thoracic Surgeons Predicted Risk of Mortality (STS-PROM) score], echocardiographic parameters left ventricular ejection fraction (EF), aortic valve (AV) peak velocity, mean transvalvular pressure gradient (PG), AV area), valve morphology (bicuspid/unicuspid/quadricuspid), and follow-up duration.

‡ Means ± SD.

† Included unicuspid, bicuspid, and quadricuspid valves.

! Days.

* Months.

# Years.

### Pooled analysis of all results

**Figure 2:**
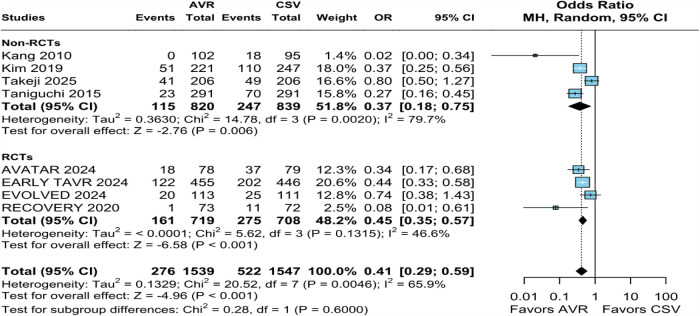
MACE AVR: aortic valve replacement; CI, confidence interval; CSV, conservative surveillance; MH, mantel-haenszel; OR, odds ratio; RCTs, randomized controlled trials.

**Figure 3:**
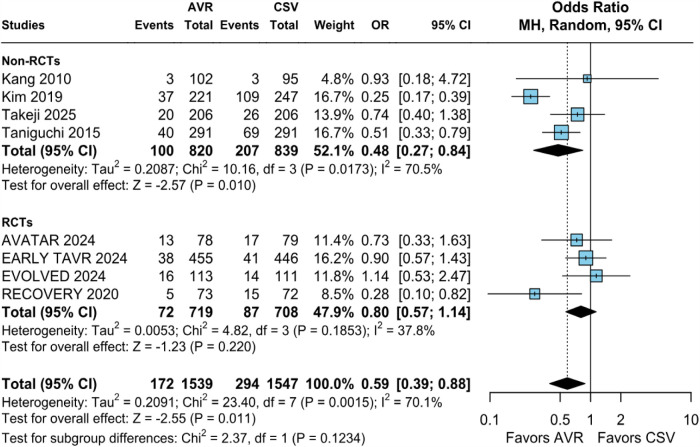
All-cause mortality AVR: aortic valve replacement; CI, confidence interval; CSV, conservative surveillance; MH, mantel-haenszel; OR, odds ratio; RCTs, randomized controlled trials.

**Figure 4:**
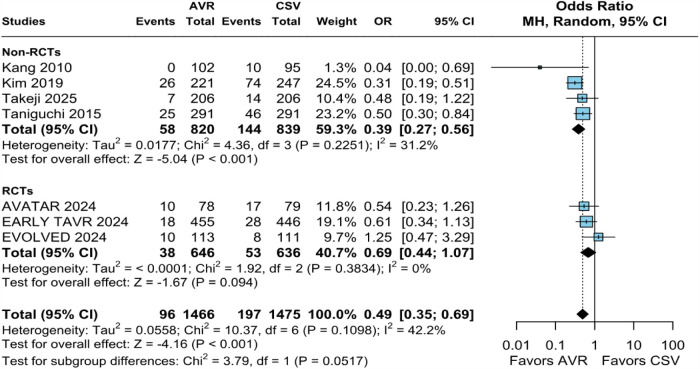
Cardiovascular mortality AVR: aortic valve replacement; CI, confidence interval; CSV, conservative surveillance; MH, mantel-haenszel; OR, odds ratio; RCTs, randomized controlled trials.

**Figure 5:**
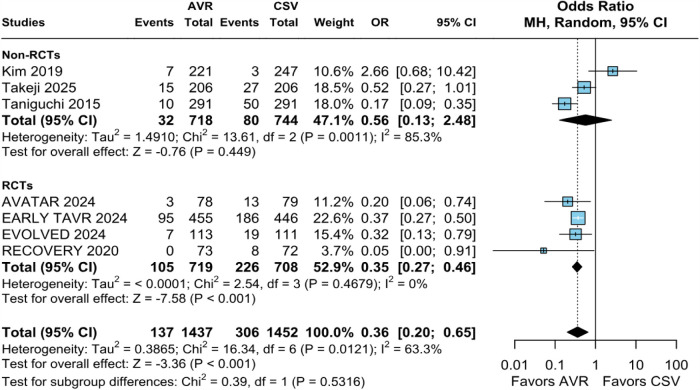
Heart failure hospitalization AVR: aortic valve replacement; CI, confidence interval; CSV, conservative surveillance; MH, mantel-haenszel; OR, odds ratio; RCTs, randomized controlled trials.

**Figure 6:**
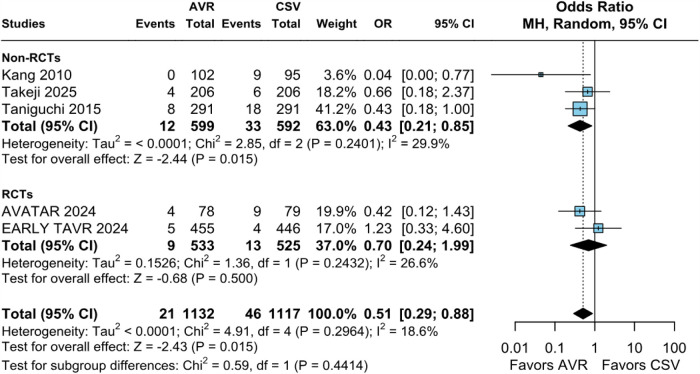
Sudden death AVR: aortic valve replacement; CI, confidence interval; CSV, conservative surveillance; MH, mantel-haenszel; OR, odds ratio; RCTs, randomized controlled trials.

**Figure 7:**
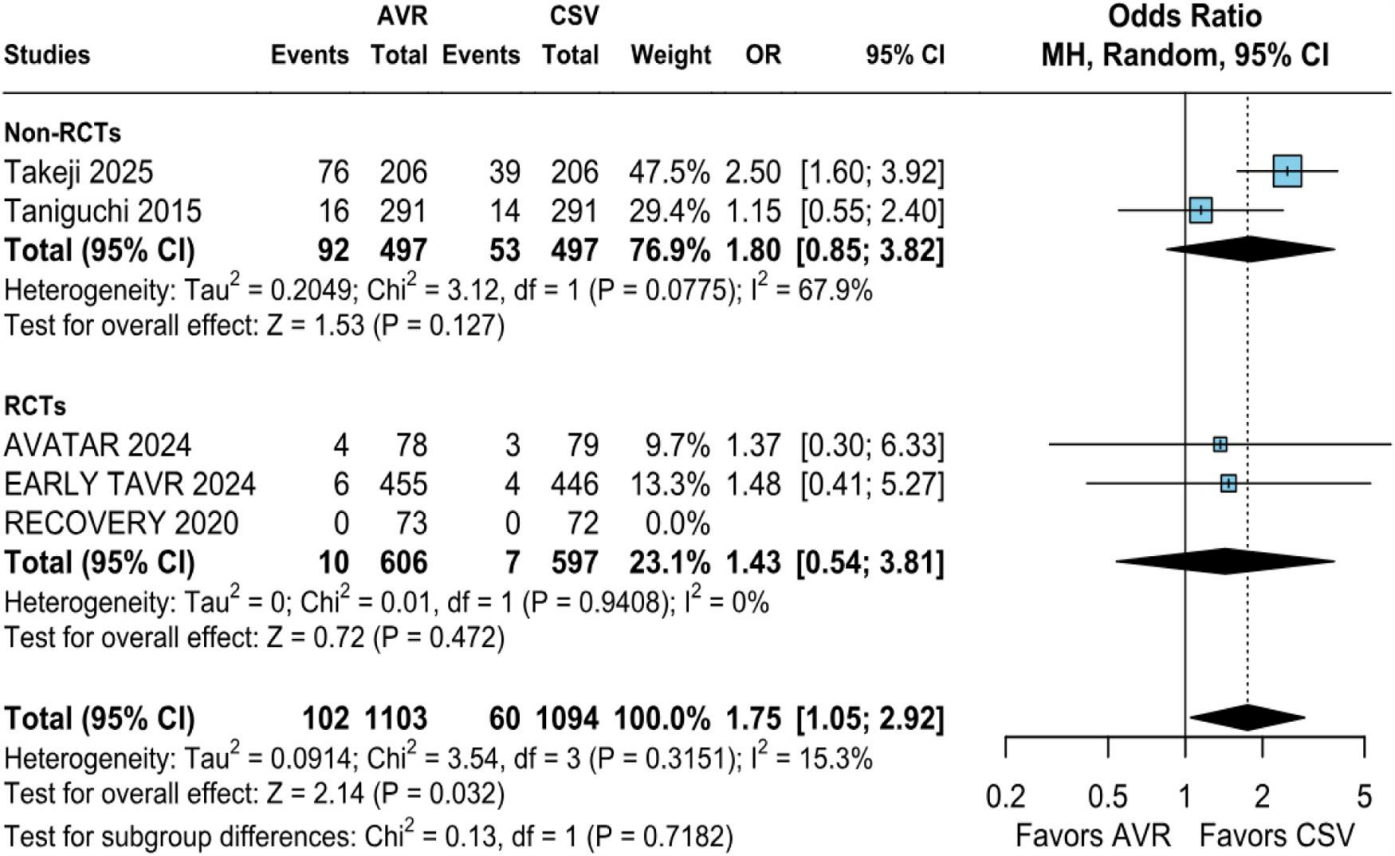
Major vascular bleeding AVR, aortic valve replacement; CI, confidence interval; CSV, conservative surveillance; MH, mantel-haenszel; OR, odds ratio; RCTs, randomized controlled trials.

There was a significant difference in the incidence of MACE (OR: 0.41; 95% CI: 0.29–0.59; *P* < 0.001; *I*^2^ = 65.9%; Figure 2), all-cause mortality (OR: 0.59; 95% CI: 0.39–0.88; *P* = 0.011; *I*^2^ = 70.1%; Figure 3), and cardiovascular mortality (OR: 0.49; 95% CI: 0.35–0.69; *P* < 0.001; *I*^2^ = 42.2%; Figure 4) between the early AVR and CSV groups. In absolute terms, MACE rates were 17.9% vs. 33.7%, corresponding to an absolute risk reduction of 15.8% and a number needed to treat of 6 (Supplementary Table S3). Furthermore, early AVR was associated with a significantly lower incidence of hospitalization for heart failure (OR, 0.36; 95% CI, 0.20–0.65; *P* < 0.001; *I*^2^ = 63.3%; Figure 5) and sudden death (OR: 0.51; 95% CI: 0.29–0.88; *P* = 0.015; *I*^2^ = 18.6%; Figure 6) than CSV in patients with asymptomatic severe AS. However, in studies that reported bleeding, the incidence of major vascular bleeding was significantly higher in the early AVR group than in the CSV group (OR: 1.75; 95% CI: 1.05–2.92; *P* = 0.032; *I*^2^ = 15.3%; Figure 7).

**Figure 8:**
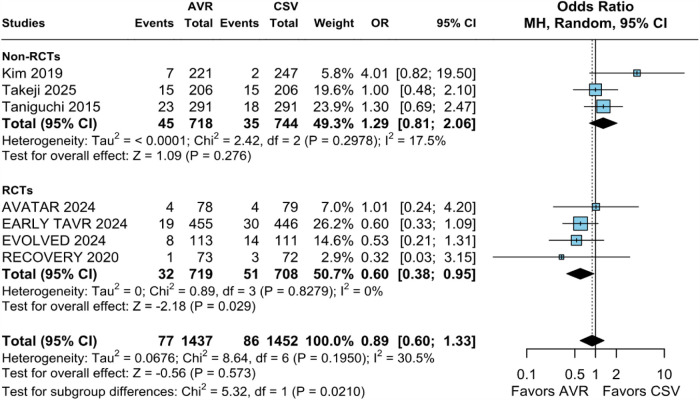
Stroke/TIA AVR: aortic valve replacement; CI, confidence interval; CSV, conservative surveillance; MH, mantel-haenszel; OR, odds ratio; RCTs, randomized controlled trials; TIA, transient ischemic attack.

**Figure 9:**
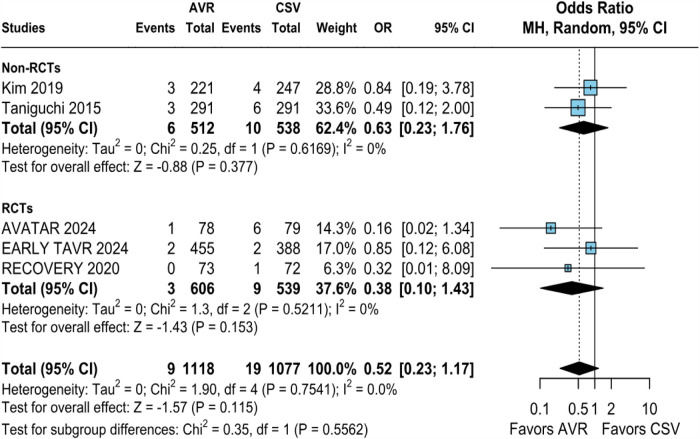
Myocardial infarction AVR, aortic valve replacement; CI, confidence interval; CSV, conservative surveillance; MH, mantel-haenszel; OR, odds ratio; RCTs, randomized controlled trials.

**Figure 10:**
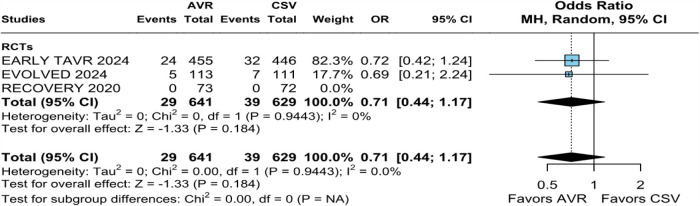
Permanent pacemaker implantation AVR, aortic valve replacement; CI, confidence interval; CSV, conservative surveillance; MH, mantel-haenszel; OR, odds ratio; RCTs, randomized controlled trials.

**Figure 11:**
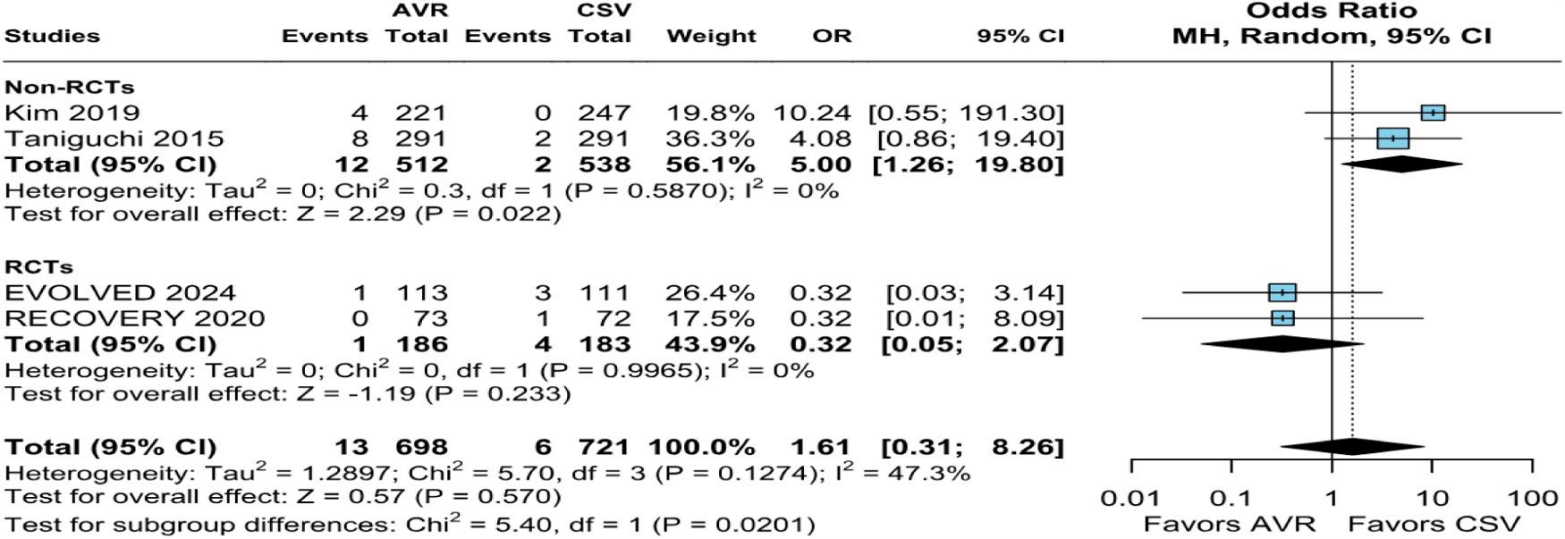
Infective endocarditis AVR: aortic valve replacement; CI: confidence interval; CSV: conservative surveillance; MH: mantel-haenszel; OR: odds ratio; RCTs: randomized controlled trials.

There were no significant differences between the groups in the incidence of stroke (OR: 0.89; 95% CI: 0.60–1.33; *P* = 0.573; *I*^2^ = 30.5%; Figure 8), MI (OR: 0.52; 95% CI: 0.23–1.17; *P* = 0.115; *I*^2^ = 0%; Figure 9), pacemaker implantation (OR: 0.71; 95% CI: 0.44–1.17; *P* = 0.184; *I*^2^ = 0%; Figure 10), and endocarditis (OR: 1.61; 95% CI: 0.31–8.26; *P* = 0.570; *I*^2^ = 47.3%; Figure 11).

**Central Illustration:**
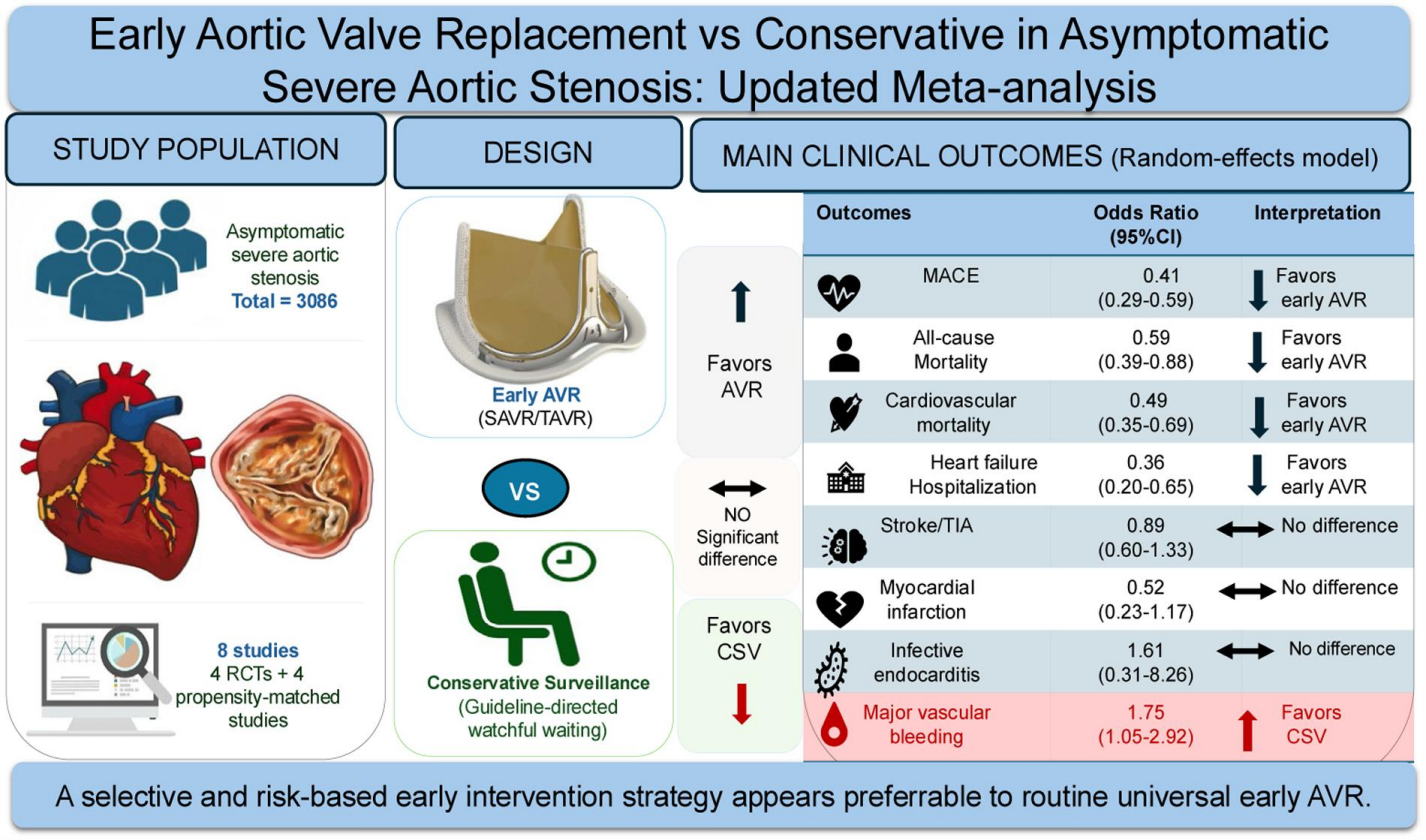
Early aortic valve replacement versus conservative surveillance in asymptomatic severe aortic stenosis. Summary of the study population, treatment strategies, and key outcomes comparing early aortic valve replacement (AVR) with conservative surveillance in asymptomatic severe aortic stenosis. Early AVR was associated with reduced major adverse cardiovascular events, mortality, and heart failure hospitalization, with no significant differences in stroke, myocardial infarction, or pacemaker implantation, but a higher risk of major vascular bleeding. These findings support a selective, risk-based approach to early intervention.

### Subgroup analyses

The prespecified subgroup analysis demonstrated consistent benefit of early AVR across study designs. For MACE, both RCTs (OR: 0.45; 95% CI: 0.35–0.57; *P* < 0.001; *I*^2^ = 46.6%; Figure 2) and observational studies (OR: 0.37; 95% CI: 0.18–0.75; *P* < 0.001; *I*^2^ = 49.7%; Figure 2) showed significant reductions, with no subgroup interaction (*P* = 0.600). All-cause mortality was not significantly reduced in RCTs (OR: 0.80; 95% CI: 0.57–1.14; *P* = 0.220; *I*^2^ = 37.8%; Figure 3) but was significantly lower in observational studies (OR: 0.48; 95% CI: 0.27–0.84; *P* = 0.010; *I*^2^ = 70.5%; Figure 3), without evidence of interaction (*P* = 0.123). Cardiovascular mortality was reduced in observational studies (OR: 0.39; 95% CI: 0.27–0.56; *P* < 0.001; *I*^2^ = 31.2%; Figure 4) but not in RCTs (OR: 0.69; 95% CI: 0.44–1.07; *P* = 0.094; *I*^2^ = 0%; Figure 4), with a suggestive difference between subgroups (*P* = 0.052).

For hospitalization for heart failure, early AVR was associated with benefit in RCTs (OR: 0.35; 95% CI: 0.27–0.46; *P* < 0.001; *I*^2^ = 0%; Figure 5) but not in observational studies (OR: 0.56; 95% CI: 0.13–2.48; *P* = 0.449; *I*^2^ = 85.3%; Figure 5), with no subgroup interaction (*P* = 0.532). Sudden death was not significantly reduced in RCTs (OR: 0.70; 95% CI: 0.24–1.99; *P* = 0.500; *I*^2^ = 18.6%; Figure 6) but was lower in observational studies (OR: 0.43; 95% CI: 0.21–0.85; *P* = 0.015; *I*^2^ = 29.9%; Figure 6), with no interaction (*P* = 0.441).

Major vascular bleeding was comparable between groups in both RCTs (OR: 1.43; 95% CI: 0.54–3.81; *P* = 0.472; *I*^2^ = 15.3%; Figure 7) and observational studies (OR: 1.80; 95% CI: 0.85–3.82; *P* = 0.472; *I*^2^ = 67.9%; Figure 7), with no subgroup difference (*P* = 0.718). Notably, stroke outcomes differed by study design. Randomized trials demonstrated a reduction in stroke with early intervention (OR: 0.60; 95% CI: 0.36–0.95), whereas observational studies showed a non-significant trend toward higher stroke risk (OR: 1.29; 95% CI: 0.81–2.05), with a significant subgroup interaction (p for interaction = 0.021). Myocardial infarction rates were similar in both RCTs (OR: 0.38; 95% CI: 0.10–1.43; *P* = 0.153; *I*^2^ = 0%; Figure 9) and observational studies (OR: 0.63; 95% CI: 0.23–1.76; *P* = 0.377; *I*^2^ = 0%; Figure 9), with no interaction (*P* = 0.556).

Finally, for infective endocarditis, RCTs showed no difference (OR: 0.32; 95% CI: 0.05–2.07; *P* = 0.233; *I*^2^² = 0%; Figure 11), whereas observational studies demonstrated a higher incidence with early AVR (OR: 5.00; 95% CI: 1.26–19.80; *P* = 0.022; *I*^2^ = 0%; Figure 11), with evidence of subgroup interaction (*P* = 0.020). Subgroup analyses stratified by procedural modality (SAVR-only vs. TAVR-including studies) demonstrated a consistent direction of effect across groups (Supplementary Table S5). Formal subgroup analyses based on MACE definitions, asymptomatic status, and disease severity were not feasible due to limited study numbers and heterogeneity in reporting; these factors are summarized in Supplementary Table S2.

Figures 2–11 Early AVR vs. clinical surveillance in patients with asymptomatic severe AS. There was a significant reduction in the incidence of MACE (OR: 0.41, 95% CI: 0.29–0.59; *P* < 0.001), all-cause mortality (OR: 0.59, 95% CI: 0.39–0.88; *P* = 0.011), cardiovascular mortality (OR: 0.49, 95% CI: 0.35–0.69; *P* < 0.001), hospitalization for heart failure (OR: 0.36, 95% CI: 0.20–0.65; *P* < 0.001), and sudden death (OR: 0.51, 95% CI: 0.29–0.88; *P* = 0.015) in patients treated with early AVR compared with CSV, whereas major vascular bleeding was significantly more in patients on CSV compared with early AVR (OR: 1.75, 95% CI: 1.05–2.92; *P* = 0.032). (1) There was no difference between groups in the incidence of myocardial infarction, stroke, permanent pacemaker implantation, and infective endocarditis. (2) MACE. (3) All-cause mortality. (4) Cardiovascular mortality. (5) Hospitalization for heart failure. (6) Sudden death. (7) Major vascular complication. (8) Stroke/TIA. (9) Myocardial infarction. (10) Permanent pacemaker. (11) Infective endocarditis.

### Sensitivity analysis

Sensitivity analyses using RRs yielded findings consistent in direction and statistical significance with the primary OR-based analyses across outcomes, supporting the robustness of the chosen effect measure (Supplementary Table S4). Additional *post hoc* sensitivity analyses using HRs, where published time-to-event estimates were available, also demonstrated concordant results. Early AVR was associated with lower hazards of MACE (HR: 0.50, 95% CI: 0.34–0.73; *P* = 0.0003; *I*^2^ = 71.6%), all-cause mortality (HR: 0.68, 95% CI: 0.54–0.86; *P* = 0.0014; *I*^2^ = 21.6%), cardiovascular mortality (HR: 0.56, 95% CI: 0.43–0.73; *P* < 0.0001; *I*^2^ = 0%), and heart failure hospitalization (HR: 0.35, 95% CI: 0.23–0.53; *P* < 0.0001; *I*^2^ = 53.8%) compared with conservative surveillance. These findings further support the consistency of benefit associated with early intervention (Supplementary Table S5).

Th leave-one-out analyses was also performed on all outcomes as a diagnostic tool to identify influential studies contributing to heterogeneity, with results illustrated for MACE, all-cause mortality, cardiovascular mortality, and heart failure hospitalization (Supplementary Fig. S1–S4). For all-cause mortality, exclusion of the study by Kim et al. reduced heterogeneity from *I*^2^ = 70.1% to *I*^2^ = 22.0%, with a corresponding change in Cochran's Q-test from *P* = 0.001 to *P* = 0.261, with the pooled effect remaining significant (OR: 0.70, 95% CI: 0.52–0.93; *P* = 0.015). Similarly, for heart failure hospitalization, heterogeneity decreased from *I*^2^ = 63.4% (*P* = 0.012) to *I*^2^ = 36.9% (*P* = 0.160) after excluding Kim et al., with the pooled effect estimate remaining significant (OR: 0.31, 95% CI: 0.21–0.46; *P* < 0.001). These findings indicate that Kim et al. contributed substantially to between-study variability; however, the reduction in I² alone does not imply that it is the sole source of variability, and results should be interpreted cautiously. For all other outcomes, the sequential removal of individual studies did not cause major alterations in the pooled effect sizes or heterogeneity. A sensitivity analysis restricted to standardized MACE definitions was not feasible due to inconsistent reporting across studies; however, this variability is summarized in Supplementary Table S2 and considered in the interpretation.

### Quality assessment

The appraisal of RCTs is summarized in Supplementary Table S6. All RCTs were open-label, with patients and operators unblinded to treatment assignment; however, outcome assessment was adjudicated independently, and overall risk of bias was judged to be low across domains. For the observational studies, propensity score matching was performed to balance baseline characteristics between intervention and control groups. Nevertheless, given their non-randomized design and the potential for residual confounding, all observational studies were deemed to have a moderate risk of bias (Supplementary Table S7). Funnel plots for MACE, all-cause mortality, cardiovascular mortality, and hospitalization for heart failure demonstrated a symmetrical distribution of studies, with point estimates converging toward the pooled effect size as study weight increased. There was no evidence of publication bias based on Begg's rank correlation test and Egger's regression test across the evaluated outcomes (Supplementary Fig. S5–S8). Publication bias tests were interpreted with caution due to the limited number of included studies. The results of the GRADE assessment are summarized in Supplementary Table S8.

### Meta-regression analysis

Meta-regression analyses were performed to explore potential sources of heterogeneity across outcomes. Study design (randomized vs. observational) and procedural modality (SAVR vs. TAVR vs. mixed cohorts) were not identified as significant moderators for any outcome, including MACE, all-cause mortality, and heart failure hospitalization (all QM *P* > 0.05), and did not explain between-study heterogeneity (*R*^2^ = 0% in most analyses). In contrast, follow-up duration emerged as a significant moderator for both MACE (QM = 3.92, *P* = 0.048; *R*^2^ = 29.4%) and all-cause mortality (QM = 4.32, *P* = 0.038; *R*^2^ = 56.1%), with longer follow-up associated with greater treatment benefit. This effect was not observed for heart failure hospitalization (QM = 0.01, *P* = 0.93; *R*^2^ = 0%). Despite these findings, residual heterogeneity remained moderate to substantial across most models (Supplementary Table S9).

## Discussion

In this systematic review and meta-analysis of eight studies with 3,086 patients, an early AVR was compared with CSV in asymptomatic patients with severe AS. The main findings were as follows: (i) early AVR was associated with approximately 2.4-fold lower odds of MACE; (ii) a significant reduction in all-cause and cardiovascular mortality, as well as sudden cardiac death; (iii) a significant reduction in hospitalization for heart failure; (iv) a higher rate of major vascular bleeding in the early AVR group; and (v) no significant differences in the incidence of MI, stroke, permanent pacemaker implantation, or infective endocarditis compared with CSV (Central illustration). Notably, GRADE assessment demonstrated moderate certainty for major efficacy outcomes, including MACE, mortality, and heart failure hospitalization, and low to very low certainty for less frequent safety outcomes, largely due to imprecision, heterogeneity, and limited study-level data.

The management of patients with asymptomatic severe AS is still guided by a Class IIa recommendation in major international guidelines, reflecting ongoing equipoise regarding the optimal timing of intervention. However, accumulating evidence suggests that AVR may be superior to CSV, with benefits extending to both hard clinical endpoints and hospitalization for heart failure. A recent meta-analysis reported that aortic valve intervention was associated with a 44% reduction in the risk of MACE compared to CSV in this population (12). Similarly, another meta-analysis demonstrated that AVR was associated with a significantly lower incidence of MACE than conservative management (25). The results of our meta-analysis are consistent with these findings, showing that AVR confers a robust reduction in MACE.

Early AVR has consistently been shown to reduce both all-cause and cardiovascular mortality in patients with asymptomatic severe AS (8, 9, 12, 13, 26–28). Although the mechanisms underlying the survival benefit are not clear, they may be related to a reduction in the risk of sudden cardiac death, prevention of progressive left ventricular remodeling and interstitial fibrosis, restoration of coronary hemodynamics, and mitigation of arrhythmic burden. In the RECOVERY trial that recruited patients with very severe asymptomatic severe AS, surgical AVR demonstrated a significant reduction in all-cause mortality compared with watchful waiting in patients with very severe AS (8). Similarly, patients with asymptomatic severe AS with preserved ejection fraction and negative exercise stress test in the AVATAR study showed that surgical AVR in asymptomatic severe AS led to a lower composite endpoint of death, myocardial infarction, stroke, or unplanned hospitalization for heart failure compared with conservative surveillance (9). Prior meta-analyses have further confirmed these benefits, reporting that AVR significantly reduces all-cause and cardiovascular mortality compared with clinical surveillance (12, 13, 26–28). Our meta-analysis corroborates these findings, demonstrating that AVR was associated with significant reductions in all-cause and cardiovascular mortality mainly due to observational studies compared with clinical surveillance. These findings highlight the ongoing clinical equipoise regarding early intervention in truly asymptomatic patients. Also, the neutral findings of the EVOLVED trial warrant cautious interpretation. The delay in aortic valve replacement within the intervention arm likely attenuated the intended pre-emptive strategy, reducing separation between early intervention and surveillance. In addition, biomarker- and imaging-guided selection, lower-than-expected event rates, and relatively short follow-up may have further limited the ability to detect differences in clinical outcomes. Accordingly, the absence of a significant benefit should be viewed in the context of these trial-specific factors rather than as definitive evidence against early intervention.

We also found a significant reduction in the incidence of hospitalization for heart failure with AVR compared with CSV. Heart failure events are critical markers of disease progression in patients with asymptomatic severe AS and are strongly associated with subsequent mortality. Therefore, prevention of the first hospitalization for heart failure is pivotal, as hospitalization not only reflects irreversible decompensation but also portends poor long-term outcomes (29–32). In the AVATAR trial, early surgical AVR significantly reduced the risk of hospitalization for heart failure compared to conservative management in patients with asymptomatic severe AS (9). Similarly, observational studies have consistently shown that patients with asymptomatic severe AS who were managed with early intervention strategy experienced lower rates of hospitalization for heart failure by approximately two-thirds of patients with asymptomatic severe AS than those undergoing watchful waiting (10, 11, 14, 17). Furthermore, prior meta-analyses that included observational studies for quantitative analysis, albeit with fewer patients, also showed significant differences in hospitalization for heart failure, further reinforcing our finding in real-world situations (13, 33).

Sudden cardiac death has been reported in up to 7.2% (0.3%–1.4% per year) of patients with asymptomatic severe AS (2, 10, 34, 35). Our meta-analysis found a pooled incidence of 1.9% in the early AVR group compared to 4.2% in the CSV group, with a statistically significant difference between strategies. This finding is clinically meaningful, as sudden death in this population is unpredictable and often occurs in patients who were previously considered to be at low risk (2, 10, 34). AVR likely helps mitigate the risks of valvular obstruction, restoring coronary hemodynamics, and reducing arrhythmic burden, thereby offering protection against sudden death, even in the absence of symptoms. Notably, sensitivity analyses using published HRs demonstrated concordant reductions in MACE, all-cause mortality, cardiovascular mortality, and heart failure hospitalization. These findings are clinically important because time-to-event analyses incorporate both event occurrence and duration of follow-up, supporting a sustained benefit of early intervention over surveillance.

The current ESC and ACC/AHA guidelines adopt a conservative approach to asymptomatic severe AS, recommending intervention only in the presence of specific high-risk features such as reduced LVEF (<50%), very severe AS (Vmax ≥5.0 m/s), abnormal exercise test, or markedly elevated B-type natriuretic peptide (6, 7). While these recommendations are designed to avoid unnecessary procedural risks, our findings suggest that AVR may offer benefits beyond these earlier defined indications. Given the reduction in procedural risks associated with SAVR and TAVR, and the demonstrated adverse outcomes associated with CSV, the threshold for recommending early intervention may need to be revisited. Incorporating refined risk stratification tools, such as cardiac magnetic resonance-detected diffuse myocardial fibrosis and midwall late gadolinium enhancement, echocardiographic global longitudinal strain, or elevated biomarkers such as BNP and high-sensitivity troponin, into guideline frameworks may better identify asymptomatic patients with severe AS who are at heightened risk under conservative management (36–38).

A critical consideration in interpreting studies of asymptomatic severe AS is the accurate classification of patients along two fundamental dimensions: “whether individuals are truly asymptomatic” and “the disease is truly severe”. Misclassification in either domain may substantially influence observed outcomes and contribute to heterogeneity across studies (35, 39). Symptom status is often assessed clinically and may be limited by patient adaptation, reduced physical activity, or underreporting, while objective evaluation with exercise testing is not uniformly performed across studies (2, 35). Consequently, some patients that were categorized as asymptomatic may in fact have exertional symptoms or latent functional limitation. Similarly, although echocardiographic criteria for severe AS are well established, variability in measurement, loading conditions, and the presence of discordant grading may lead to misclassification of disease severity (6, 7). Emerging approaches incorporating multimodality imaging, such as cardiac magnetic resonance-derived myocardial fibrosis assessment, as well as circulating biomarkers, may further help refine risk stratification and identify patients at higher risk despite the absence of overt symptoms (15, 36–38). These considerations are particularly relevant in observational studies, where standardized protocols for symptom assessment and disease severity may be less rigorously applied compared with randomized trials.

In this study, significant heterogeneity was observed across major endpoints, including MACE, all-cause mortality, and heart failure hospitalization. Leave-one-out analysis identified the study by Kim et al. as a major contributor to heterogeneity across key outcomes, with a marked reduction in I² and a shift from statistically significant to non-significant heterogeneity following its exclusion. This disproportionate influence on heterogeneity may likely reflect differences in patient classification, particularly the non-routine use of exercise testing, which may have resulted in inclusion of patients who were not truly asymptomatic. This highlights the importance of rigorous phenotyping in studies of asymptomatic severe AS. Exploratory meta-regression analyses did not identify a single dominant source of heterogeneity, likely reflecting limited statistical power given the small number of included studies. Instead, heterogeneity is more plausibly explained by cumulative differences in study design, procedural modality, follow-up duration, and patient selection. RCTs enrolled rigorously selected patients with standardized assessment of asymptomatic status, whereas observational studies reflected broader real-world populations, introducing potential selection bias and unmeasured confounding. Subgroup analyses stratified by procedural modality demonstrated a consistent direction of effect, suggesting that intervention type alone is unlikely to account for the observed variability. In addition, variability in the assessment of symptom status and disease severity across studies may have contributed to differences in observed outcomes, particularly in observational cohorts. Differences in follow-up duration may also have influenced outcome variability, as longer follow-up tends to accentuate divergence in clinical events between early intervention and conservative management. Consistent with this, meta-regression analyses did not identify study design or procedural modality as significant sources of heterogeneity across outcomes, whereas follow-up duration emerged as the only consistent moderator for MACE and all-cause mortality, suggesting a time-dependent treatment effect whereby the benefits of early intervention may accrue over time. This association was not observed for heart failure hospitalization, indicating that this outcome may be influenced by additional clinical or methodological factors not captured in study-level analyses. In additional exploratory meta-regression analyses for heart failure hospitalization, proportion of females, confirmation of truly asymptomatic status, and inclusion of patients with very severe AS were not significant moderators of treatment effect. These findings suggest that the residual heterogeneity in HF hospitalization is unlikely to be attributable to a single measured study-level factor and more likely reflects cumulative differences in hospitalization thresholds, local practice patterns, patient comorbidity burden, and post-procedural management strategies that were incompletely reported across studies. Sensitivity analyses using RRs yielded consistent findings across all outcomes, supporting the robustness of the primary effect estimates. While ORs may overestimate effect sizes when event rates are not rare, the concordance between OR- and RR-based analyses supports the validity of the observed associations. Subgroup interaction tests remain inherently underpowered, and borderline findings should be interpreted cautiously; similarly, *I*^2^ values in the lower range should not be assumed to indicate true homogeneity, particularly in analyses with few studies. Overall, residual heterogeneity remained moderate to substantial, reinforcing the likelihood of multifactorial influences and underscoring the need for cautious interpretation given the inherent limitations of study-level meta-regression.

While early AVR in asymptomatic severe AS is associated with clinical benefits, these must be balanced against procedural risks. Surgical AVR may be complicated by bleeding, stroke, infection, and atrial fibrillation, whereas transcatheter approaches carry risks such as vascular injury, pacemaker implantation, and access-site bleeding (40–42). However, contemporary data suggest that complication rates have declined with advances in patient selection, device technology, and procedural expertise (43, 44). Overall, subgroup and sensitivity analyses indicate that the net clinical benefit of early intervention remains favorable, although decisions should be individualized, weighing short-term procedural risks against long-term outcomes.

The discordant findings for stroke between randomized and observational studies warrant careful consideration. Randomized trials demonstrated a reduction in stroke with early intervention (8, 9, 16), whereas observational studies showed a non-significant trend in the opposite direction (10, 11, 14, 17), with a significant subgroup interaction. This discrepancy likely reflects differences in patient selection, procedural modality, and temporal evolution of practice. Observational cohorts may include earlier-generation transcatheter procedures associated with higher periprocedural embolic risk, whereas contemporary trials reflect improved techniques and operator experience (4, 5, 42, 43). Additionally, differences in the proportion of surgical vs. transcatheter valve replacement across studies may further contribute to this variation (11, 15, 17). As such, the overall pooled estimate may mask important differences across study designs and should be interpreted cautiously. The increased incidence of infective endocarditis observed in observational studies also merits attention. This likely reflects the risk of prosthetic valve endocarditis, a recognized complication of both surgical and transcatheter valve replacement, associated with substantial morbidity and mortality (39–41, 45). Although absolute event rates were low, this remains clinically relevant particularly in younger or lower-risk patients in whom lifetime exposure to valve-related complications and durability concerns influence the timing of intervention (42, 43). While this signal was not observed in randomized trials and should be interpreted cautiously given the small number of events and wide confidence intervals, it underscores the importance of incorporating long-term infection risk into shared decision-making.

These findings support a selective early intervention strategy in appropriately chosen patients. Early AVR provides substantial reductions in major adverse cardiovascular events and mortality (NNT 6–15), with minimal impact on stroke, myocardial infarction, and pacemaker implantation, but at the cost of increased procedural complications, particularly major vascular bleeding (NNH ≈ 27).

### Impact on clinical practice

This study integrates contemporary randomized and propensity-matched evidence to inform the optimal timing of pre-emptive AVR in asymptomatic severe AS. Early intervention was associated with reductions in MACE, mortality, and heart failure hospitalization, without a meaningful impact on stroke or myocardial infarction, supporting a more individualized approach to management. By restricting inclusion to randomized and propensity-matched studies, incorporating structured assessment of whether patients were truly asymptomatic and truly severe, and evaluating procedural safety outcomes including pacemaker implantation, infective endocarditis, and major vascular complications, this analysis provides a balanced benefit-risk framework.

These findings complement current ESC and ACC/AHA guideline recommendations, which support AVR in selected asymptomatic patients with severe AS who exhibit high-risk features such as very severe stenosis, reduced left ventricular ejection fraction, abnormal exercise testing, rapid hemodynamic progression, markedly elevated natriuretic peptides, or evidence of adverse cardiac remodeling. Importantly, however, the populations included in this meta-analysis were not restricted to patients fulfilling these contemporary high-risk criteria. Accordingly, the observed reductions in MACE, mortality, and heart failure hospitalization suggest that pre-emptive AVR may also confer benefit in selected asymptomatic severe AS patients who do not yet meet conventional guideline triggers for intervention.

Nevertheless, these findings should not be interpreted as support for universal early intervention in all asymptomatic severe AS patients. Rather, they support refinement of current guideline-directed management through individualized risk stratification, multidisciplinary heart-team evaluation, and earlier consideration of AVR in selected patients with markers of adverse remodeling, disease progression, or increased cardiovascular risk. These results have important implications for patient selection, procedural decision-making, and shared decision-making, while underscoring the need for further adequately powered randomized trials to better define which asymptomatic patients derive the greatest benefit from pre-emptive intervention.

## Limitations

This study has several limitations. First, a key methodological limitation is the heterogeneity in the definition of MACE across included studies. Components ranged from hard endpoints such as death, myocardial infarction, and stroke to broader outcomes including heart failure or cardiovascular hospitalization, and in some cases AVR. The inclusion of valve intervention as a MACE component may structurally favor the clinical surveillance arm, whereas its exclusion may bias results toward early intervention. Accordingly, the pooled MACE estimate reflects a composite outcome with variable clinical meaning and should be interpreted cautiously. Second, approximately half of the included studies were observational, introducing potential selection bias and residual confounding despite the use of propensity matching. While subgroup and sensitivity analyses demonstrated consistent direction of effect, the absence of a clear mortality benefit in randomized trials highlights that the observed survival signal in pooled analyses is influenced by non-randomized data and should be interpreted with caution. Third, residual heterogeneity across outcomes likely reflects multifactorial differences in study design, procedural modality, follow-up duration, and patient selection, including variability in the classification of patients as truly asymptomatic and truly severe, female representation, prevalence of very severe AS, and other incompletely reported clinical or procedural characteristics. Although exploratory meta-regression identified follow-up duration as a moderator for MACE and all-cause mortality, substantial unexplained heterogeneity remained, particularly for heart failure hospitalization. Although heterogeneity was explored through subgroup and exploratory analyses, these factors may not be fully accounted for. Fourth, although ORs were used as the primary effect measure, sensitivity analyses using RRs yielded consistent findings, mitigating concerns regarding potential overestimation when event rates were not rare. Additional sensitivity analyses using HRs should be interpreted cautiously, as HRs were not uniformly reported across all studies or outcomes, and the covariates included in adjusted models varied between trials. Accordingly, these analyses were exploratory and intended to complement, rather than replace, the primary pooled estimates. Fifth, follow-up duration varied across studies, limiting assessment of long-term outcomes, including valve durability particularly for transcatheter approaches. Finally, the overall sample size may have been insufficient to detect small but clinically meaningful differences in less frequent outcomes such as stroke and sudden cardiac death.

## Conclusions

This meta-analysis, involving 3,086 patients with asymptomatic severe AS, demonstrates that a pre-emptive AVR strategy is associated with reductions in MACE and heart failure hospitalization compared with clinical surveillance. Although pooled analyses suggest a potential survival benefit, this was not consistently observed in randomized trials and appears to be driven largely by observational data; therefore, it should be interpreted cautiously. Further large-scale randomized trials with longer follow-up are needed to clarify the impact on survival, valve durability, and stroke outcomes, and to better inform patient selection and guideline recommendations. Future studies incorporating advanced analytic approaches, including Bayesian frameworks, may further refine risk stratification and clinical decision-making in this population.

## Data Availability

The original contributions presented in the study are included in the article/Supplementary Material, further inquiries can be directed to the corresponding author.

## References

[1] Santangelo G Bursi F Faggiano A Moscardelli S Simeoli P Guazzi M. The global burden of valvular heart disease: from clinical epidemiology to management. J Clin Med. (2023) 12(6):2178. 10.3390/jcm12062178 36983180 PMC10054046

[2] Pellikka PA Sarano ME Nishimura RA Malouf JF Bailey KR Scott CG. Outcome of 622 adults with asymptomatic, hemodynamically significant aortic stenosis during prolonged follow-up. Circulation. (2005) 111(24):3290–5. 10.1161/CIRCULATIONAHA.104.495903 15956131

[3] Heuvelman HJ van Geldorp MWA Kappetein AP Geleijnse ML Galema TW Bogers AJJC. Clinical course of patients diagnosed with severe aortic stenosis in the rotterdam area: insights from the AVARIJN study. Neth Heart J. (2012) 20(12):487–93. 10.1007/s12471-012-0309-3 22864980 PMC3515726

[4] Popma JJ Deeb GM Yakubov SJ Mumtaz M Gada H O’Hair D. Transcatheter aortic-valve replacement with a self-expanding valve in low-risk patients. N Engl J Med. (2019) 380(18):1706–15. 10.1056/NEJMoa1816885 30883053

[5] Forrest JK Deeb GM Yakubov SJ Rovin JD Mumtaz M Gada H. 2-Year Outcomes after transcatheter versus surgical aortic valve replacement in low-risk patients. J Am Coll Cardiol. (2022) 79(9):882–96. 10.1016/j.jacc.2021.11.062 35241222

[6] Praz F Borger MA Lanz J Marin-Cuartas M Abreu A Adamo M. ESC/EACTS scientific document group, 2025 ESC/EACTS guidelines for the management of valvular heart disease: developed by the task force for the management of valvular heart disease of the European Society of Cardiology (ESC) and the European association for cardio-thoracic surgery (EACTS). Eur Heart J. (2025) 46:4635–736. 10.1093/eurheartj/ehaf194 40878295

[7] Otto CM Nishimura RA Bonow RO Carabello BA Erwin JP Gentile F. 2020 ACC/AHA guideline for the management of patients with valvular heart disease: executive summary: a report of the American college of cardiology/American heart association joint committee on clinical practice guidelines. Circulation. (2021) 143(5):e35–71. 10.1161/CIR.0000000000000932 33332149

[8] Kang D-H Park S-J Lee S-A Lee S Kim D-H Kim H-K. Early surgery or conservative care for asymptomatic aortic stenosis. N Engl J Med. (2020) 382(2):111–9. 10.1056/NEJMoa1912846 31733181

[9] Banovic M Putnik S Da Costa BR Penicka M Deja MA Kotrc M. Aortic valve replacement vs. Conservative treatment in asymptomatic severe aortic stenosis: long-term follow-up of the AVATAR trial. Eur Heart J. (2024) 45(42):4526–35. 10.1093/eurheartj/ehae585 39217448

[10] Kang D-H Park S-J Rim JH Yun S-C Kim D-H Song J-M. Early surgery versus conventional treatment in asymptomatic very severe aortic stenosis. Circulation. (2010) 121(13):1502–9. 10.1161/CIRCULATIONAHA.109.909903 20308614

[11] Taniguchi T Morimoto T Shiomi H Ando K Kanamori N Murata K. CURRENT AS registry investigators. Initial surgical versus conservative strategies in patients with asymptomatic severe aortic stenosis. J Am Coll Cardiol. (2015) 66(25):2827–38. 10.1016/j.jacc.2015.10.001 26477634

[12] Song Q Liu R Yang K Tu X Tan H Fan C. Early aortic valve replacement of asymptomatic severe aortic stenosis: a meta-analysis of randomized controlled trials. J Am Heart Assoc. (2025) 14(16):e041283. 10.1161/JAHA.125.041283 40831305 PMC12533610

[13] de Pontes VB Clemente MRC Trevisan T Jaramillo S Boneli MF Felix N. Early aortic-valve replacement in patients with asymptomatic severe aortic stenosis with preserved left ventricular systolic function: a systematic review and meta-analysis. Am J Cardiol. (2025) 248:73–9. 10.1016/j.amjcard.2025.03.039 40180138

[14] Kim HJ Kim JB Kim HR Ju MH Kang D-Y Lee S-A. Impact of valve replacement on long-term survival in asymptomatic patients with severe aortic stenosis. Am J Cardiol. (2019) 123(8):1321–8. 10.1016/j.amjcard.2019.01.035 30745019

[15] Loganath K Craig NJ Everett RJ Bing R Tsampasian V Molek P. EVOLVED Investigators. Early intervention in patients with asymptomatic severe aortic stenosis and myocardial fibrosis: the EVOLVED randomized clinical trial. JAMA. (2025) 333(3):213–21. 10.1001/jama.2024.22730 39466640 PMC11519785

[16] Généreux P Schwartz A Oldemeyer JB Pibarot P Cohen DJ Blanke P. EARLY TAVR trial investigators. Transcatheter aortic-valve replacement for asymptomatic severe aortic stenosis. N Engl J Med. (2025) 392(3):217–27. 10.1056/NEJMoa2405880 39466903

[17] Takeji Y Taniguchi T Morimoto T Shirai S Kitai T Tabata H. CURRENT AS registry-2 investigators. Early aortic valve replacement versus clinical surveillance in asymptomatic patients with severe aortic stenosis. Am J Cardiol. (2025) 253:68–77. 10.1016/j.amjcard.2025.06.004 40490131

[18] Page MJ McKenzie JE Bossuyt PM Boutron I Hoffmann TC Mulrow CD. The PRISMA 2020 statement: an updated guideline for reporting systematic reviews. Br Med J. (2021) 1:89. 10.1136/bmj.n71 PMC8008539 33781348

[19] Higgins JPT Thomas J Chandler J Cumpston M Li T Page MJ. Cochrane Handbook for Systematic Reviews of Interventions Version 6.5 (updated August 2024). Cochrane. Chichester: John Wiley & Sons (2024). Available online at: www.cochrane.org/handbook

[20] Sterne JA Hernán MA Reeves BC Savović J Berkman ND Viswanathan M. ROBINS-I: a tool for assessing risk of bias in non-randomised studies of interventions. Br Med J. (2016) 355:i4919. 10.1136/bmj.i4919 27733354 PMC5062054

[21] Sterne JAC Savović J Page MJ Elbers RG Blencowe NS Boutron I. Rob 2: a revised tool for assessing risk of bias in randomised trials. Br Med J. (2019) 366:l4898. 10.1136/bmj.l4898 31462531

[22] Mcguinness LA Higgins JPT. Risk-of-bias VISualization (robvis): an R package and shiny web app for visualizing risk-of-bias assessments. Res Synth Methods. (2021) 12(1):55–61. 10.1002/jrsm.1411 32336025

[23] Luo D Wan X Liu J Tong T. Optimally estimating the sample mean from the sample size, median, mid-range, and/or mid-quartile range. Stat Methods Med Res. (2018) 27(6):1785–805. 10.1177/0962280216669183 27683581

[24] Wan X Wang W Liu J Tong T. Estimating the sample mean and standard deviation from the sample size, median, range and/or interquartile range. BMC Med Res Methodol. (2014) 14:135. 10.1186/1471-2288-14-135 25524443 PMC4383202

[25] Senguttuvan NB Srinivasan NV Panchanatham M Abdulkader RS Anandaram A Polareddy DR. Systematic review and meta-analysis of early aortic valve replacement versus conservative therapy in patients with asymptomatic aortic valve stenosis with preserved left ventricle systolic function. Open Heart. (2024) 11(1):e002511. 10.1136/openhrt-2023-002511 38191233 PMC10806528

[26] Ferlini M Munafò AR Lanzillo G Aiello M Gazzoli F Mirizzi AM. Early surgical aortic valve replacement in asymptomatic patients with severe aortic stenosis: a systematic review and meta-analysis. J Cardiovasc Med. (2022) 23(9):632–4. 10.2459/JCM.0000000000001338 35905003

[27] Costa GNF Cardoso JFL Oliveiros B Gonçalves L Teixeira R. Early surgical intervention versus conservative management of asymptomatic severe aortic stenosis: a systematic review and meta-analysis. Heart. (2023) 109(4):314–21. 10.1136/heartjnl-2022-321411 36198484

[28] Brar SK Leong DW Razi RR Moore N Zadegan R Mansukhani P. Early aortic valve replacement in asymptomatic severe aortic stenosis: a meta-analysis of randomized controlled trials. Am J Cardiol. (2025) 245:11–6. 10.1016/j.amjcard.2025.02.025 40054514

[29] Jalava MP Laakso T Virtanen M Niemelä M Ahvenvaara T Tauriainen T. Transcatheter and surgical aortic valve replacement in patients with recent acute heart failure. Ann Thorac Surg. (2020) 109(1):110–7. 10.1016/j.athoracsur.2019.05.044 31288017

[30] Kaewkes D Ochiai T Flint N Patel V Patel J Kim I. Transcatheter aortic valve implantation in patients with severe aortic stenosis hospitalized with acute heart failure. Am J Cardiol. (2021) 144:100–10. 10.1016/j.amjcard.2020.12.046 33383005

[31] Thilén M James S Ståhle E Lindhagen L Christersson C. Pre-operative heart failure worsens outcome after aortic valve replacement irrespective of left ventricular ejection fraction. Eur Heart J Qual Care Clin Outcomes. (2022) 8(2):127–34. 10.1093/ehjqcco/qcab008 33543245

[32] Fukutomi M Onishi T Ando T Higuchi R Hagiya K Saji M. Impact of prior hospitalization for heart failure on clinical outcomes of patients after transcatheter aortic valve implantation with new-generation devices: insights from the LAPLACE-TAVI registry. Catheter Cardiovasc Interv. (2024) 104(7):1469–76. 10.1002/ccd.31261 39402889

[33] Ismayl M Machanahalli Balakrishna A Abusnina W Thandra A Walters RW Alugubelli NR. Surgical aortic valve replacement versus conservative treatment in asymptomatic severe aortic stenosis: an updated systematic review and meta-analysis. Cardiovasc Revasc Med. (2022) 42:36–44. 10.1016/j.carrev.2022.03.001 35292208

[34] Rosenhek R Binder T Porenta G Lang I Christ G Schemper M. Predictors of outcome in severe, asymptomatic aortic stenosis. N Engl J Med. (2000) 343(9):611–6117. 10.1056/NEJM200008313430903 10965007

[35] Généreux P Stone GW O’Gara PT Marquis-Gravel G Redfors B Giustino G. Natural history, diagnostic approaches, and therapeutic strategies for patients with asymptomatic severe aortic stenosis. J Am Coll Cardiol. (2016) 67(19):2263–88. 10.1016/j.jacc.2016.02.057 27049682

[36] Lee H-J Singh A Lim J Craig N Bing R Tastet L. Diffuse interstitial fibrosis of the myocardium predicts outcome in moderate and asymptomatic severe aortic stenosis. JACC Cardiovasc Imaging. (2025) 18(2):180–91. 10.1016/j.jcmg.2024.08.003 39340492

[37] Yingchoncharoen Y Gibby C Rodriguez LL Grimm RA Marwick TH. Association of myocardial deformation with outcome in asymptomatic aortic stenosis with normal ejection fraction. Circ Cardiovasc Imaging. (2012) 5(6):719–25. 10.1161/CIRCIMAGING.112.977348 23008423

[38] White M Baral R Ryding A Tsampasian V Ravindrarajah T Garg P. Biomarkers associated with mortality in aortic stenosis: a systematic review and meta-analysis. Med Sci. (2021) 9(2):29. 10.3390/medsci9020029 PMC8163007 34067808

[39] Karampinos K Ktenopoulos N Apostolos A Koliastasis L Kachrimanidis I Vlachakis P. Navigating the silence: reconsidering treatment paradigms in asymptomatic severe aortic stenosis. Hellenic J Cardiol. (2026) 87:101–20. 10.1016/j.hjc.2025.06.004 40532874

[40] Koo HJ Lee HN Anh TT Kang J-W Yang DH Song J-K. Postoperative complications after surgical aortic valve replacement. Cardiovasc Imaging Asia. (2017) 1(4):222–30. 10.22468/cvia.2017.00115

[41] Arnold SV Zhang Y Baron SJ McAndrew TC Alu MC Kodali SK. Impact of short-term complications on mortality and quality of life after transcatheter aortic valve replacement. JACC Cardiovas Interv. (2019) 12(4):362–9. 10.1016/j.jcin.2018.11.008 PMC6392020 30784641

[42] Culler SD Reynolds MR Kugelmass AD Katz MR Simon AW Cohen DJ. Incremental cost and length of stay associated with complications of transcatheter aortic valve replacement. JACC Adv. (2025) 4(9):102107. 10.1016/j.jacadv.2025.102107 40865191 PMC12409306

[43] Lauck SB Baron SJ Irish W Borregaard B Moore KA Gunnarsson CL. Temporal changes in mortality after transcatheter and surgical aortic valve replacement: retrospective analysis of US medicare patients (2012–2019). J Am Heart Assoc. (2021) 10(20):e021748. 10.1161/JAHA.120.021748 34581191 PMC8751862

[44] Harvey JE Kapadia SR Cohen DJ Kalra A Irish W Gunnarsson C. Trends in complications among patients undergoing aortic valve replacement in the United States. J Am Heart Assoc. (2024) 13(17):e031461. 10.1161/JAHA.123.031461 39189613 PMC11646526

[45] Summers MR Leon MB Smith CR Kodali SK Thourani VH Herrmann HC. Prosthetic valve endocarditis after TAVR and SAVR. Circulation. (2019) 140(24):1984–94. 10.1161/CIRCULATIONAHA.119.041399 31690104

