# Supplementary material for: Early aortic valve intervention versus clinical surveillance in patients with asymptomatic severe aortic stenosis: a systematic review and meta-analysis
Source: Front Cardiovasc Med. 2026 Jun 23;13:1830856. doi: 10.3389/fcvm.2026.1830856 (PMC13337704; doi:10.3389/fcvm.2026.1830856)
Supplement: Supplementary file 1 [file Datasheet1.pdf]

Supplementary information

Umar G. Adamu, Confidence Makgoro, El-ameen U Adamu, David Mashilo, Anupa Patel, Nqoba Tsabedze

Supplementary Table S1 Search strategies for all the electronic databases

Supplementary Table S2 Definition of asymptomatic severe aortic stenosis and MACE

Supplementary Table S3 Absolute risk differences and numbers needed to treat or harm

Supplementary Table S4 Sensitivity analysis using risk ratios of the outcomes

Supplementary Table S5 Sensitivity analysis using hazard ratios of the outcomes

Supplementary Table S6 Risk of Bias assessment for RCTs

Supplementary Table S7 Risk of Bias assessment for observational studies

Supplementary Table S8 GRADE assessment profile

Supplementary Table S9 Meta-regression analyses of study-level factors associated with treatment effects across major outcomes

Supplementary Fig. S1 Leave-one-out analysis of MACE

Supplementary Fig. S2 Leave-one-out analysis of all-cause mortality

Supplementary Fig. S3 Leave-one-out analysis of cardiovascular mortality

Supplementary Fig. S4 Leave-one-out analysis of hospitalization for heart failure

Supplementary Fig. S5 Funnel plot of MACE

Supplementary Fig. S6 Funnel plot of all-cause mortality

Supplementary Fig. S7 Funnel plot of cardiovascular mortality

Supplementary Fig. S8 Funnel plot of hospitalization for heart failure

Supplementary Table S1 Search strategies for all the electronic databases

**PubMed**	("Aortic stenosis"[Mesh] OR "aortic valve stenosis" OR "aortic valve stenosis"[Mesh] OR "aortic valve disease" OR "severe aortic stenosis") AND (Asymptomatic OR "asymptomatic diseases"[Mesh] OR "no symptoms" OR "minimally symptomatic") AND ("Early intervention" OR "early surgery" OR "early aortic valve replacement" OR "early AVR" OR "preemptive surgery" OR "timely intervention" OR "aortic valve replacement"[Mesh] OR TAVR OR TAVI OR "transcatheter aortic valve replacement" OR "Transcatheter Aortic Valve Replacement"[Mesh] OR "surgical aortic valve replacement")
**Embase**	("Aortic stenosis" OR "aortic valve stenosis" OR "aortic valve disease" OR "severe aortic stenosis") AND (Asymptomatic OR "no symptoms" OR "minimally symptomatic") AND ("Early intervention" OR "early surgery" OR "early aortic valve replacement" OR "early AVR" OR "preemptive surgery" OR "timely intervention" OR TAVR OR TAVI OR SAVR OR "transcatheter aortic valve replacement" OR "surgical aortic valve replacement")
**Cochrane**	("Aortic stenosis" OR "aortic valve stenosis" OR "aortic valve disease" OR "severe aortic stenosis") AND (Asymptomatic OR "no symptoms" OR "minimally symptomatic") AND ("Early intervention" OR "early surgery" OR "early aortic valve replacement" OR "early AVR" OR "preemptive surgery" OR "timely intervention" OR TAVR OR TAVI OR SAVR OR "transcatheter aortic valve replacement" OR "surgical aortic valve replacement")
**Scopus**	("Aortic stenosis" OR "aortic valve stenosis" OR "aortic valve disease" OR "severe aortic stenosis") AND (Asymptomatic OR "no symptoms" OR "minimally symptomatic") AND ("Early intervention" OR "early surgery" OR "early aortic valve replacement" OR "early AVR" OR "preemptive surgery" OR "timely intervention" OR TAVR OR TAVI OR SAVR OR "transcatheter aortic valve replacement" OR "surgical aortic valve replacement") AND (PUBYEAR < 2026)
**Web of Science**	("Aortic stenosis" OR "aortic valve stenosis" OR "aortic valve disease" OR "severe aortic stenosis") AND (Asymptomatic OR "no symptoms" OR "minimally symptomatic") AND ("Early intervention" OR "early surgery" OR "early aortic valve replacement" OR "early AVR" OR "preemptive surgery" OR "timely intervention" OR TAVR OR TAVI OR SAVR OR "transcatheter aortic valve replacement" OR "surgical aortic valve replacement")

Supplementary Table S2 Definition of asymptomatic severe aortic stenosis and MACE

Studies	Definition of severe aortic stenosis	Asymptomatic	MACE definition	Truly Asymptomatic?	Truly Severe?
AVATAR 2024	Vmax across the aortic valve > 4.0 m/s, or Pmean ≥ 40 mmHg and AVA ≤ $1 cm^2$ or AVAi ≤ $0.6 cm^2/m^2$ at rest,	No reported symptoms (exercise testing performed in trial protocol)	All-cause mortality or acute myocardial infarction, stroke, and unplanned heart failure hospitalization	Yes, objective assessment with stress testing	Yes, guideline consistent
EARLY TAVR 2024	AVA ≤$1.0 cm^2$ or AVAi ≤$0.6 cm^2/m^2$ AND Vmax ≥4.0 m/s or Pmean ≥40 mmHg	Negative treadmill stress test and must not demonstrate any of the following during and/or after the test: Syncopal or pre-syncopal episode, including severe dizziness, angina, limiting dyspnea or decreased exercise tolerance, defined as inability to reach 60% of age and sex adjusted metabolic equivalents of task drop in systolic blood pressure (defined as a progressive drop of at least 20 mmHg and sustained for 1 minute or an acute drop of 40 mmHg), significant ventricular arrhythmias (≥4 consecutive ventricular premature beats) OR per physician after thorough assessment of patient history if the patient is unable to perform a stress test.	Death from any cause, stroke, or unplanned hospitalization for cardiovascular causes.	Yes, rigorous objective confirmation	Yes, strict hemodynamic criteria
EVOLVED 2024	Vmax ≥4.0 m/s, or AVAi <$0.6cm^2/m^2$ Vmax ≥3.5m/s	No symptoms attributable to aortic stenosis that require aortic valve replacement	All-cause mortality or unplanned aortic stenosis–related hospitalization.	Probably, clinical only, no systematic stress testing	Yes, in addition to imaging-based refinement
Kang 2010	Very severe aortic stenosis AVA ≤0.75 cm^2, Vmax ≥4.5 m/s or Pmean ≥ 50 mmHg	No reported symptoms, no routine exercise testing	Operative mortality and cardiac death during follow-up.	Uncertain, no stress testing	Yes, very severe AS
Kim 2019	AVA ≤$1.0 cm^2$ or AVAi ≤$0.6 cm^2/m^2$ AND Vmax ≥4.0 m/s or mean gradient ≥40 mmHg	No reported symptoms<br>However, exercise tests are not routinely performed	Nonfatal myocardial infarction, stroke, infective endocarditis, hospitalization for heart failure, and AV reoperation.	Uncertain, limited objective assessment	Yes, guideline consistent
RECOVERY 2019	Very severe aortic stenosis AVA ≤0.75 cm^2, Vmax ≥4.5 m/s or Pmean ≥ 50 mmHg	No exertional dyspnea, syncope, presyncope, angina, ejection fraction <50% and negative exercise test	Operative mortality or death from cardiovascular causes during follow-up.	Yes, objective confirmation	Yes, very severe AS
Takeji 2025	Very severe aortic stenosis AVA ≤$1.0 cm^2$ or Vmax ≥4.0 m/s or Pmean ≥ 40 mmHg	Negative exercise and/or a 6-minute walk test	All-cause death, stroke or hospitalization for heart failure.	Yes, functional testing included	Yes, guideline consistent
Taniguchi 2015	Very severe aortic stenosis AVA ≤$1.0 cm^2$, Vmax ≥4.0 m/s and Pmean ≥ 40 mmHg	Not clearly stated	All-cause death and heart failure hospitalization.	Uncertain, not clearly defined	Yes, guideline consistent

AS: Aortic stenosis; AV: aortic valve; AVA: Aortic valve area; AVAi: Aortic valve area index; MACE: major adverse cardiac events; Pmean = mean aortic pressure gradient; Vmax = peak velocity

Supplementary Table S3 Absolute risk differences and numbers needed to treat or harm

Outcomes	Early AVR	CSV	Absolute reduction	NNT/NNH
**MACE**	17.9	33.7	-15.8	NNT= 6
**ACM**	11.2	19.0	-7.8	NNT = 13
**CVM**	6.6	13.4	-6.8	NNT = 15
**HFH**	9.5	21.1	-11.6	NNT = 9
**Stroke**	5.4	5.9	-0.56	NNT = 179
**Myocardial infarction**	0.8	1.8	-0.95	NNT = 105
**Sudden death**	1.9	4.1	-2.3	NNT = 44
**PPM**	4.5	6.2	-1.9	NNT = 60
**Infective endocarditis**	1.9	0.8	+1.03	NNH = 97
**Major vascular bleeding**	9.3	5.5	+3.8	NNH = 27

ACM: all-cause mortality; AVR: aortic valve replacement; CVM: cardiovascular mortality; CSV: clinical surveillance; HFH: heart failure hospitalization; NNH: number needed to harm; NNT: number needed to harm; PPM: permanent pacemaker implantation

Absolute risks were calculated using pooled event counts across included studies. These estimates are approximate and should be interpreted with caution given the use of aggregated study-level data. Negative values indicate absolute risk reduction (favoring early AVR), whereas positive values indicate absolute risk increase (harm).

Supplementary Table S4 Sensitivity analysis using risk ratios of all outcomes

Outcome	Study design	Risk ratio	95% CI	p-value	I^2 (%)
**MACE**	Non-RCTs	0.47	0.26-0.83	0.010	80.0
	RCTs	0.59	0.50-0.69	<0.001	40.5
	**Total**	**0.54**	**0.41-0.69**	**<0.001**	**66.0**
**All-cause mortality**	Non-RCTs	0.55	0.37-0.79	0.002	52.9
	RCTs	0.83	0.62-1.12	0.222	34.7
	**Total**	**0.65**	**0.48-0.88**	**0.006**	**61.0**
**Cardiovascular mortality**	Non-RCTs	0.45	0.34-0.60	<0.001	19.3
	RCTs	0.71	0.47-1.06	0.093	0.0
	**Total**	**0.53**	**0.41-0.69**	**<0.001**	**32.6**
**Heart failure hospitalization**	Non-RCTs	0.60	0.15-2.37	0.465	84.4
	RCTs	0.41	0.27-0.63	<0.001	26.5
	**Total**	**0.42**	**0.24-0.71**	**0.001**	**64.9**
**Sudden death**	Non-RCTs	0.44	0.23-0.86	0.016	26.8
	RCTs	0.70	0.27-1.86	0.476	22.1
	**Total**	**0.52**	**0.31-0.88**	**0.015**	**14.5**
**Major vascular bleeding**	Non-RCTs	1.64	1.00-2.67	0.049	45.2
	RCT	1.42	0.55-3.68	0.473	0.0
	**Total**	**1.66**	**1.15-2.38**	**0.006**	**0.0**
**Stroke/TIA**	Non-RCTs	1.26	0.82-1.95	0.288	18.6
	RCTs	0.62	0.40-0.95	0.030	0.0
	**Total**	**0.90**	**0.62-1.30**	**0.558**	**29.9**
**Myocardial infarction**	Non-RCTs	0.63	0.23-1.74	0.378	0.0
	RCTs	0.39	0.11-1.43	0.155	0.0
	**Total**	**0.53**	**0.24-1.17**	**0.117**	**0.0**
**Permanent pacemaker implantation**	RCT	0.73	0.46-1.16	0.184	0.0
**Infective endocarditis**	Non-RCTs	4.89	1.25-19.11	0.022	0.0
	RCTs	0.33	0.05-2.06	0.234	0.0
	**Total**	**1.60**	**0.32-8.03**	**0.571**	**47.1**

CI: confidence interval; MACE: major adverse cardiovascular events; Non-RCTs: non-randomized controlled trials; RCTs: randomized controlled trials; TIA: transient ischemic attack.

Legnd: Risk ratios (RRs) were calculated as a sensitivity analysis to assess the robustness of the primary OR-based results. Results are presented stratified by study design (randomized vs. observational) and overall pooled estimates.

Supplementary Table S5 Sensitivity analysis using hazard ratios of all outcomes

**Outcomes**	**Study design**	**Hazard ratio**	**95% CI**	**p-value**	**I^2 (%)**
**MACE**	Non-RCTs	0.51	0.18-1.40	0.192	0.0
	RCTs	0.51	0.42-0.62	<0.0001	46
	**Total**	**0.50**	**0.34-0.73**	**0.0003**	**71.6**
**All-cause mortality**	Non-RCTs	0.64	0.49-0.84	0.0010	0.0
	RCTs	0.68	0.39-1.18	0.1742	61.2
	**Total**	**0.68**	**0.54-0.86**	**0.0014**	**21.6**
**Cardiovascular mortality**	Non-RCTs	0.56	0.40-0.78	0.0005	0.0
	RCTs	0.54	0.31-0.93	0.0261	36.7
	**Total**	**0.56**	**0.43-0.73**	**<0.0001**	**0.0**
**Heart failure hospitalization**	Non-RCTs	0.32	0.10-0.99	0.0478	83.3
	RCTs	0.41	0.32-0.52	<0.0001	0.0
	**Total**	**0.35**	**0.23-0.53**	**0.0001**	**53.8**
**Major vascular bleeding**	Non-RCTs	0.11	0.54-2.32	-	-
		0.85	1.59-3.44	-	-
	**Total**	**0.32**	**0.04-2.29**	**0.2555**	**95.7**
**Stroke/TIA**	Non-RCTs	0.50	0.20-1.23	01326	66.8
	RCTs	0.65	0.41-1.02	0.0597	0.0
	**Total**	**0.56**	**0.38-0.83**	**0.0033**	**18.4**
**Myocardial infarction**	RCTs	0.43	0.10-1.85	-	-
		0.20	0.03-1.29	-	-
	**Total**	**0.32**	**0.10-1.02**	**0.0531**	**0.0**
**Infective endocarditis**	Non-RCTs	1.30	0.98-25.90	-	-
	RCTs	0.31	0.03-3.14	-	-
	**Total**	**0.82**	**0.21-3.28**	**0.7800**	**5.4**

CI: confidence interval; MACE: major adverse cardiovascular events; Non-RCTs: non-randomized controlled trials; RCTs: randomized controlled trials; TIA: transient ischemic attack.

Legnd: Hazard ratios (RRs) were calculated as a sensitivity analysis to assess the robustness of the primary OR-based results. Results are presented stratified by study design (randomized vs. observational) and overall pooled estimates.

Supplementary Table S6 Risk of Bias assessment for RCTs

Risk of bias domains

Study	D1	D2	D3	D4	D5	Overall
AVATAR 2024	+	+	+	+	+	+
EARLY TAVR 2024	+	+	+	+	+	+
EVOLVED 2024	+	+	+	+	+	+
RECOVERY 2020	+	+	+	+	+	+

Domains:
D1: Bias arising from the randomization process.
D2: Bias due to deviations from intended intervention.
D3: Bias due to missing outcome data.
D4: Bias in measurement of the outcome.
D5: Bias in selection of the reported result.

Judgement
+ Low

Supplementary Table S7 Risk of Bias assessment for observational studies

Risk of bias domains

Study	D1	D2	D3	D4	D5	D6	D7	Overall
Kang 2010	+	+	+	+	+	-	+	-
Kim 2019	+	+	+	+	+	-	+	-
Takeji 2025	+	+	+	+	+	-	+	-
Taniguchi 2015	+	+	+	+	+	-	+	-

Domains:
D1: Bias due to confounding.
D2: Bias due to selection of participants.
D3: Bias in classification of interventions.
D4: Bias due to deviations from intended interventions.
D5: Bias due to missing data.
D6: Bias in measurement of outcomes.
D7: Bias in selection of the reported result.

Judgement
- Moderate
+ Low

Supplementary Table S8 GRADE assessment profile

Early aortic valve replacement compared to Clinical surveillance for patients with asymptomatic severe aortic stenosis

Patient or population: Patients with asymptomatic severe aortic stenosis
Setting: There a both RCTs and observational studies and observational studies selected if deriving the effect
Intervention: Early aortic valve replacement
Comparison: Clinical surveillance

Outcomes	№ of participants (studies) Follow-up	Certainty of the evidence (GRADE)	Relative effect (95% CI)	Anticipated absolute effects	
				Risk with Clinical surveillance	Risk difference with Early aortic valve replacement
Major adverse cardiovascular events assessed with: n	3086 (8 RCTs)	⊕⊕⊕◯ Moderate[a]	**OR 0.41** (0.29 to 0.59)	337 per 1 000	**165 fewer per 1 000** (from 209 fewer to 106 fewer)
All-cause mortality assessed with: n	3086 (8 non-randomised studies)	⊕⊕⊕◯ Moderate[a,b]	**OR 0.59** (0.39 to 0.88)	190 per 1 000	**68 fewer per 1 000** (from 106 fewer to 19 fewer)
Cardiovascular mortality assessed with: n	2941 (7 non-randomised studies)	⊕⊕⊕◯ Moderate[b,c]	**OR 0.49** (0.35 to 0.69)	134 per 1 000	**63 fewer per 1 000** (from 82 fewer to 37 fewer)
Heart failure hospitalization assessed with: n	2889 (7 RCTs)	⊕⊕⊕◯ Moderate[a,d]	**OR 0.36** (0.27 to 0.46)	211 per 1 000	**123 fewer per 1 000** (from 143 fewer to 101 fewer)
Sudden death assessed with: n	2249 (5 non-randomised studies)	⊕⊕◯◯ Low[e,f]	**OR 0.51** (0.29 to 0.88)	41 per 1 000	**20 fewer per 1 000** (from 29 fewer to 5 fewer)
Major vascular complications assessed with: n	2197 (5 RCTs)	⊕⊕◯◯ Low[b,g,h]	**OR 1.75** (1.05 to 2.92)	55 per 1 000	**37 more per 1 000** (from 3 more to 90 more)
Stroke/Transient ischemic attack assessed with: n	2889 (7 RCTs)	⊕⊕◯◯ Low[f,i]	**OR 0.89** (0.60 to 1.33)	59 per 1 000	**6 fewer per 1 000** (from 23 fewer to 18 more)
Myocardial infarction assessed with: n	2195 (5 RCTs)	⊕⊕◯◯ Low[g,i]	**OR 0.52** (0.23 to 1.17)	18 per 1 000	**8 fewer per 1 000** (from 14 fewer to 3 more)

**Early aortic valve replacement compared to Clinical surveillance for patients with asymptomatic severe aortic stenosis**					
**Patient or population:** Patients with asymptomatic severe aortic stenosis<br>**Setting:** There a both RCTs and observational studies and observational studies selected if deriving the effect<br>**Intervention:** Early aortic valve replacement<br>**Comparison:** Clinical surveillance					
**Outcomes**	**№ of participants (studies) Follow-up**	**Certainty of the evidence (GRADE)**	**Relative effect (95% CI)**	**Anticipated absolute effects**	
				**Risk with Clinical surveillance**	**Risk difference with Early aortic valve replacement**
Permanent pacemaker implantation assessed with: n	1270 (3 RCTs)	⨁⨁◯◯ Low[g,i,j]	**OR 0.71** (0.44 to 1.17)	62 per 1 000	**17 fewer per 1 000** (from 34 fewer to 10 more)
Infective endocarditis assessed with: n	1114 (4 non-randomised studies)	⨁◯◯◯ Very low[e,k]	**OR 1.61** (0.31 to 8.26)	8 per 1 000	**5 more per 1 000** (from 6 fewer to 56 more)

*__The risk in the intervention group__ (and its 95% confidence interval) is based on the assumed risk in the comparison group and the **relative effect** of the intervention (and its 95% CI).

CI: confidence interval; **OR:** odds ratio

GRADE Working Group grades of evidence
High certainty: we are very confident that the true effect lies close to that of the estimate of the effect.
Moderate certainty: we are moderately confident in the effect estimate: the true effect is likely to be close to the estimate of the effect, but there is a possibility that it is substantially different.
Low certainty: our confidence in the effect estimate is limited: the true effect may be substantially different from the estimate of the effect.
Very low certainty: we have very little confidence in the effect estimate: the true effect is likely to be substantially different from the estimate of effect.

Explanations

a. There is significant heterogeneity with observational studies and mixed evidence base, and the effect is large and consistent
b. The observational studies have high heterogeneity (I2=70.5%) but with narrow confidence interval.
c. Mixed evidence
d. Variability in outcome definitions and hospitalization thresholds across studies
e. All the observational studies were propensity score matched but had some concerns in the measurement of outcomes.
f. Sparse events
g. Limited number of studies.
h. The observational studies were propensity score matched, associated with high heterogeneity (I2=67.9%) and wide confidence interval but was not significant.
i. Confidence interval crosses no effect
j. Small sample size.
k. Wide confidence interval

Supplementary Table S9 Meta-regression analyses of study-level factors associated with treatment effects across major outcomes

Outcome	Covariant	QM	p-value	R^2 (%)	I^2 (%)
**Major adverse cardiovascular events**	Study design	0.05	0.83	0.0	70.6
	Procedural modality	2.73	0.26	0.0	71.5
	Follow-up duration	3.92	0.048	2.94	56.3
**All-cause mortality**	Study design	1.46	0.23	31.1	59.9
	Procedural modality	0.65	0.72	0.0	71.4
	Follow-up duration	4.32	0.038	56.1	49.9
**Heart failure hospitalization**	Study design	0.95	0.33	0.0	69.1
	Procedural modality	1.48	0.48	0.0	72.4
	Follow-up duration	0.01	0.93	0.0	69.3
	Proportion of females	0.002	0.97	0.0	69.3
	Truly asymptomatic	0.17	0.68	0.0	68.6
	Very severe aortic stenosis	1.00	0.32	0.0	67.3

Meta-regression analyses assessed study-level moderators of treatment effect across major outcomes (MACE, all-cause mortality, and heart failure hospitalization), including study design, procedural modality, and follow-up duration. Results are reported as QM statistics, p-values, R^2 (explained heterogeneity), and residual I^2. Findings should be interpreted with caution given the limited number of studies.

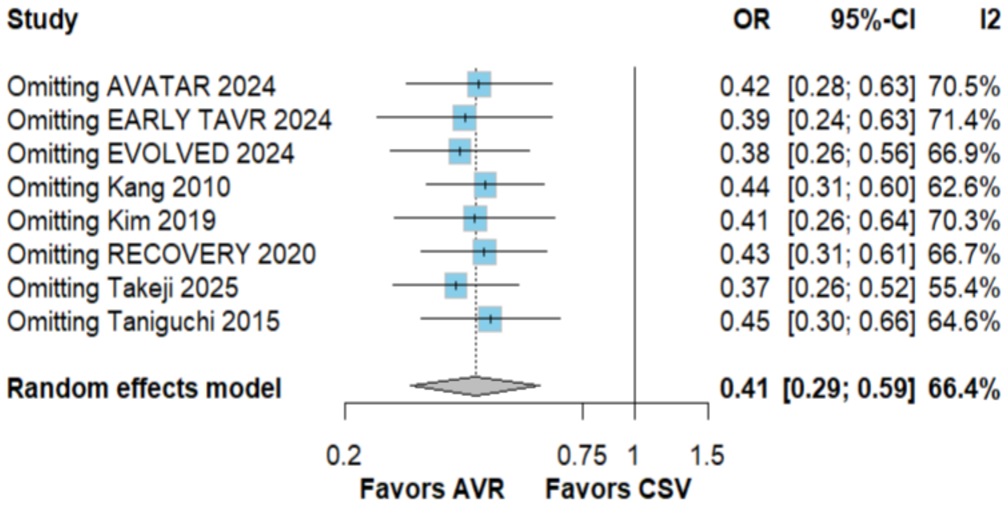

Supplementary Fig. S1 Leave-one-out analysis for MACE AVR: aortic valve replacement; CI: confidence interval; CSV: conservative surveillance; OR: odds ratio.

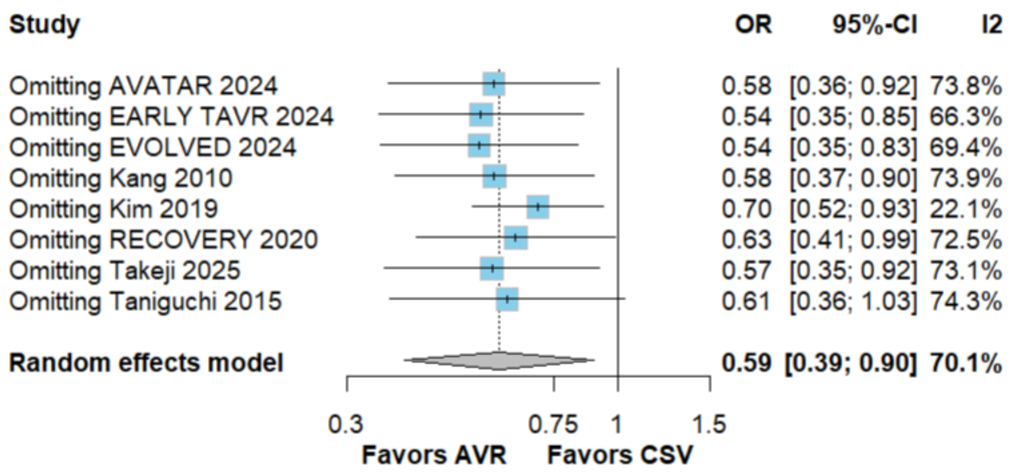

Supplementary Fig. S2 Leave-one-out analysis for all-cause mortality AVR: aortic valve replacement; CI: confidence interval; CSV: conservative surveillance; OR: odds ratio.

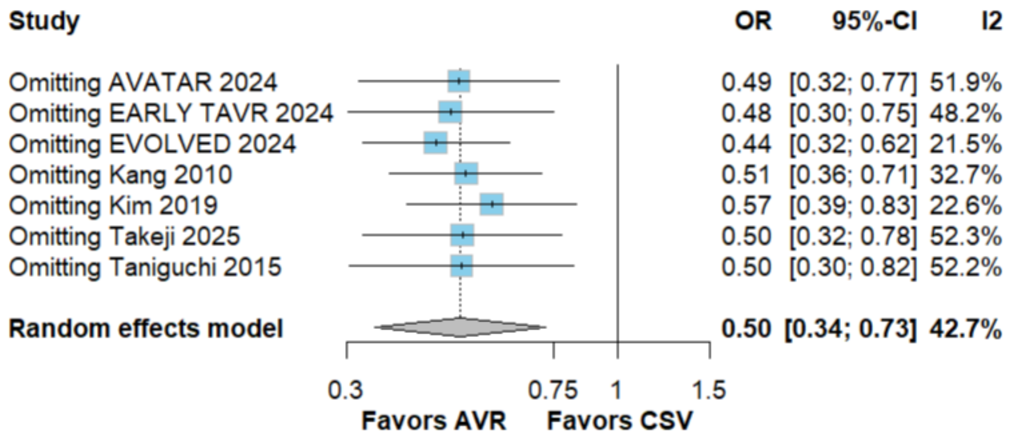

Supplementary Fig. S3 Leave-one-out analysis for cardiovascular mortality AVR: aortic valve replacement; CI: confidence interval; CSV: conservative surveillance; OR: odds ratio.

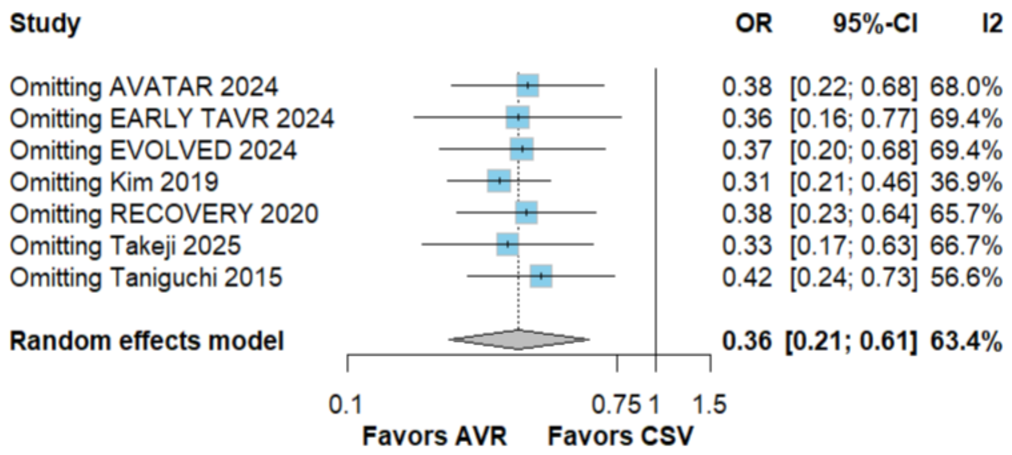

Supplementary Fig. S4 Leave-one-out analysis for hospitalization for heart failure AVR: aortic valve replacement; CI: confidence interval; CSV: conservative surveillance; OR: odds ratio.

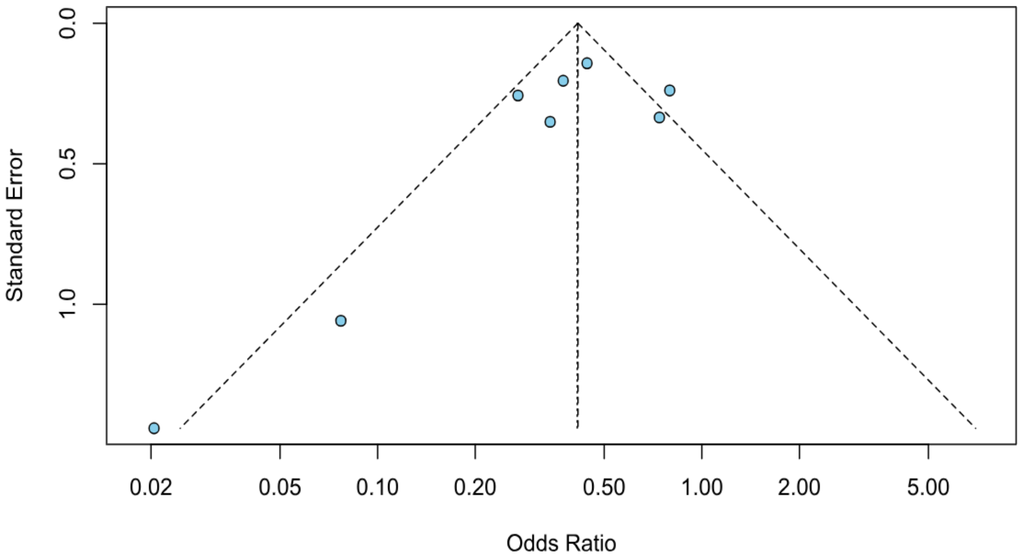

Outcome	Begg's rank correlation tau	p-value	Egger's test Intercept	95% CI	p-value
MACE	-0.43	0.18	-1.49	-3.89, -0.83	0.24

Supplementary Fig. S5 Publication bias of MACE

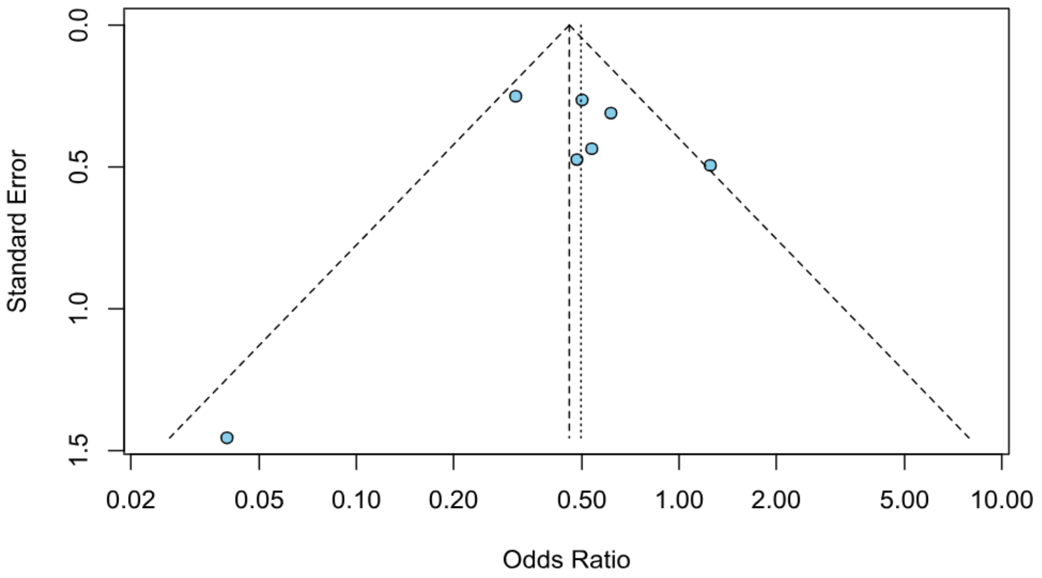

Outcome	Begg's rank correlation tau	p-value	Egger's test Intercept	95% CI	p-value
All-cause mortality	-0.07	0.90	1.23	-2.4, -4.87	0.67

Supplementary Fig. S6 Publication bias for all-cause mortality

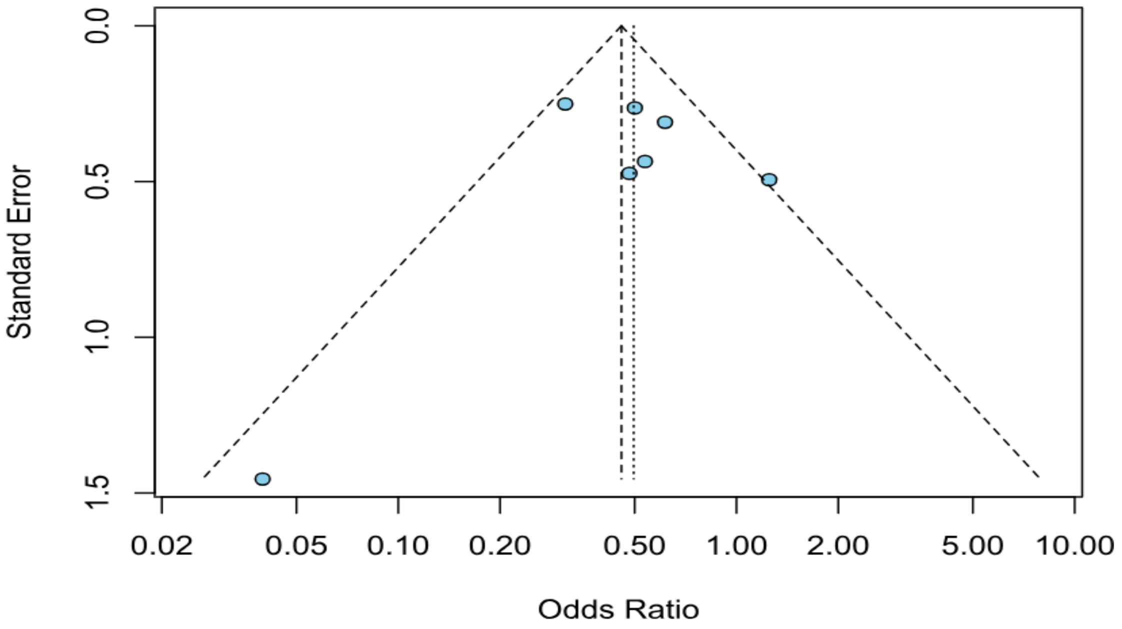

Outcome	Begg's rank correlation tau	p-value	Egger's test Intercept	95% CI	p-value
Cardiovascular mortality	0.14	0.77	-0.16	-2.94, -2.62	0.91

Supplementary Fig. S7 Publication bias for cardiovascular mortality

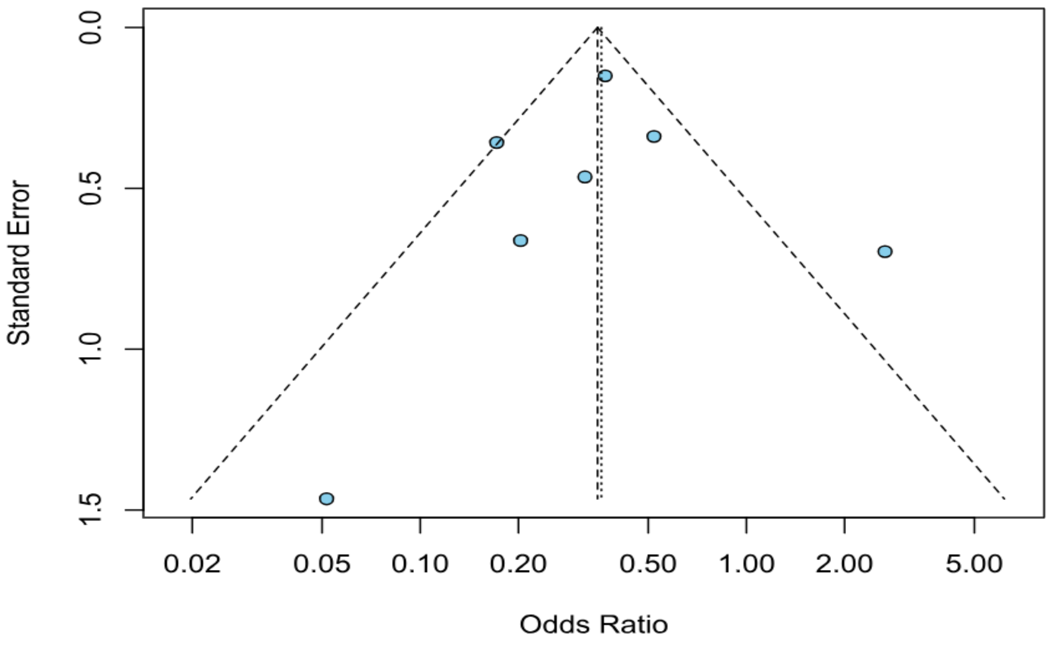

Outcome	Begg's rank correlation tau	p-value	Egger's test Intercept	95% CI	p-value
Heart failure hospitalization	-0.14	0.77	-0.16	-2.49, -2.18	0.90

Supplementary Fig. S8 Publication bias for hospitalization for heart failure

Supplementary Fig. S4 Leave-one-out analysis for hospitalization for heart failure AVR: aortic valve replacement; CI: confidence interval; CSV: conservative surveillance; OR: odds ratio.

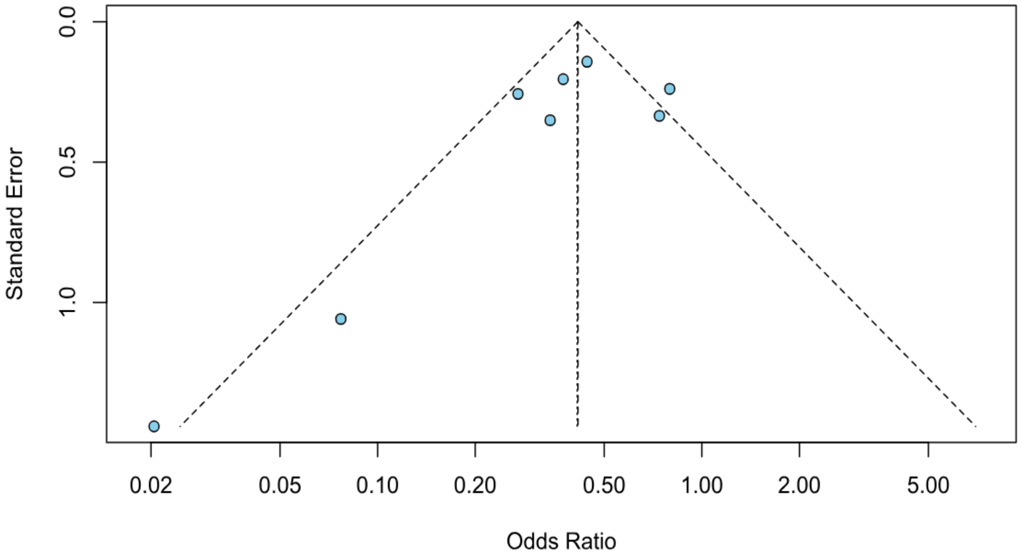

Outcome	Begg's rank correlation tau	p-value	Egger's test Intercept	95% CI	p-value
MACE	-0.43	0.18	-1.49	-3.89, -0.83	0.24

Supplementary Fig. S5 Publication bias of MACE

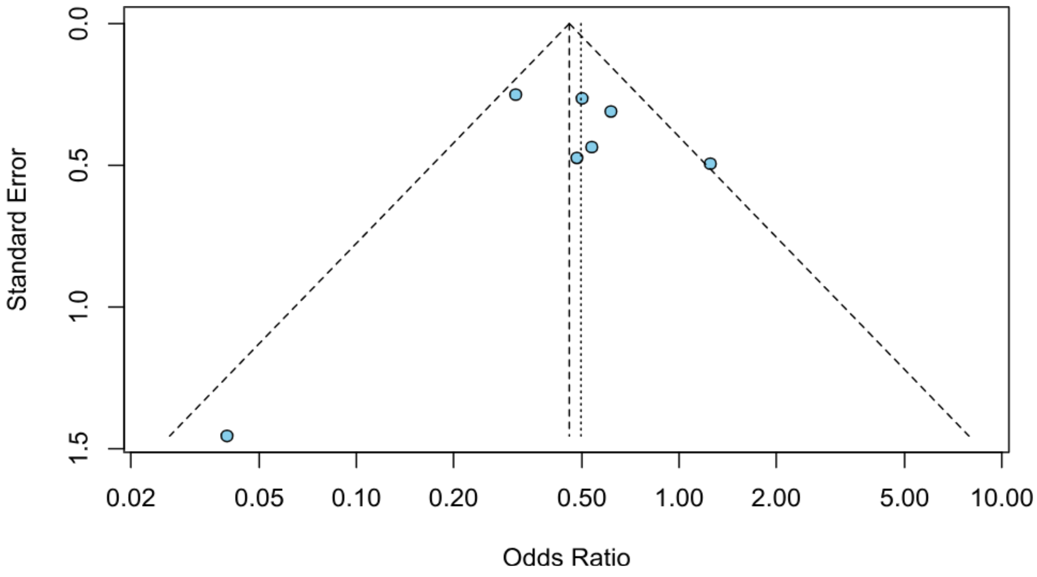

Outcome	Begg's rank correlation tau	p-value	Egger's test Intercept	95% CI	p-value
All-cause mortality	-0.07	0.90	1.23	-2.4, -4.87	0.67

Supplementary Fig. S6 Publication bias for all-cause mortality

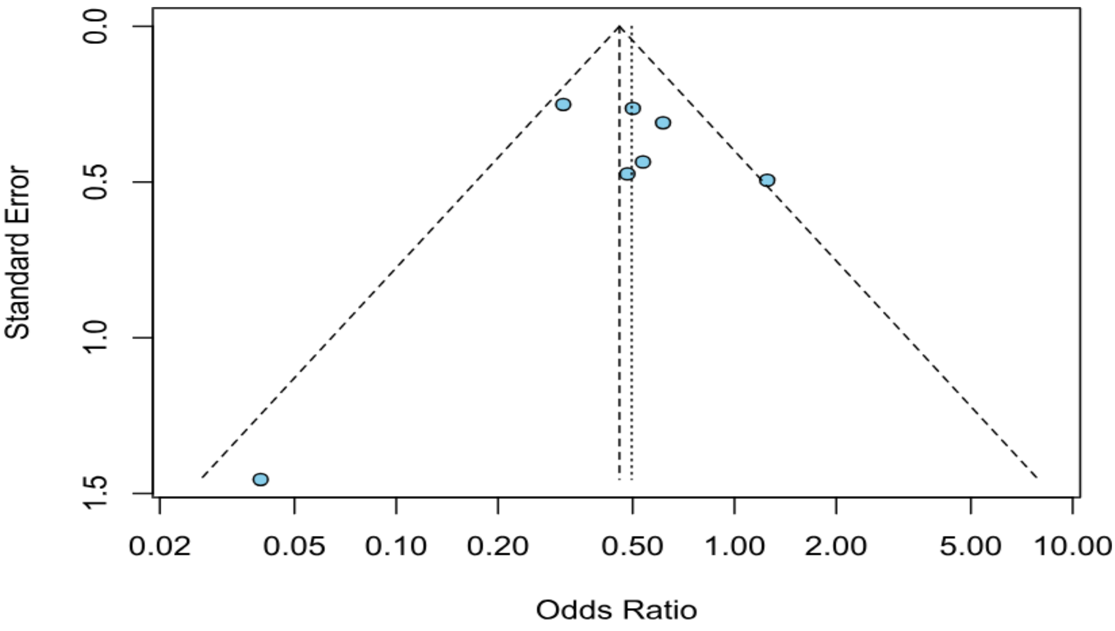

Outcome	Begg's rank correlation tau	p-value	Egger's test Intercept	95% CI	p-value
Cardiovascular mortality	0.14	0.77	-0.16	-2.94, -2.62	0.91

Supplementary Fig. S7 Publication bias for cardiovascular mortality

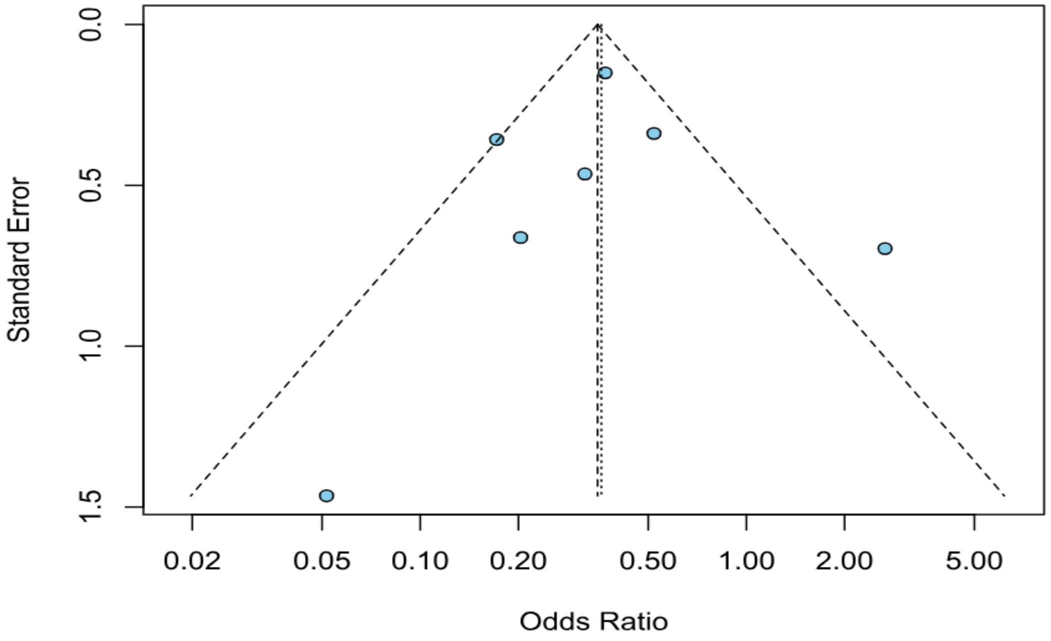

Outcome	Begg's rank correlation tau	p-value	Egger's test Intercept	95% CI	p-value
Heart failure hospitalization	-0.14	0.77	-0.16	-2.49, -2.18	0.90

Supplementary Fig. S8 Publication bias for hospitalization for heart failure
